# Application of Social Robots in Healthcare: Review on Characteristics, Requirements, Technical Solutions

**DOI:** 10.3390/s23156820

**Published:** 2023-07-31

**Authors:** Luca Ragno, Alberto Borboni, Federica Vannetti, Cinzia Amici, Nicoletta Cusano

**Affiliations:** 1 Department of Mechanical and Industrial Engineering, Università degli Studi di Brescia, Via Branze 38, 25123 Brescia, Italy; 2 IRCCS Fondazione Don Carlo Gnocchi, Via di Scandicci 269, 50143 Florence, Italy; 3 Faculty of Political Science and Sociopsychological Dynamics, Università degli Studi Internazionali, Via Cristoforo Colombo 200, 00147 Rome, Italy

**Keywords:** social robots, healthcare, telepresence robot, human–robot interaction, artificial intelligence, companion robot

## Abstract

Cyber-physical or virtual systems or devices that are capable of autonomously interacting with human or non-human agents in real environments are referred to as social robots. The primary areas of application for biomedical technology are nursing homes, hospitals, and private homes for the purpose of providing assistance to the elderly, people with disabilities, children, and medical personnel. This review examines the current state-of-the-art of social robots used in healthcare applications, with a particular emphasis on the technical characteristics and requirements of these different types of systems. Humanoids robots, companion robots, and telepresence robots are the three primary categories of devices that are identified and discussed in this article. The research looks at commercial applications, as well as scientific literature (according to the Scopus Elsevier database), patent analysis (using the Espacenet search engine), and more (searched with Google search engine). A variety of devices are enumerated and categorized, and then our discussion and organization of their respective specifications takes place.

## 1. Introduction

Social robots (SR) are artificial systems capable of playing an active and positive social role within a society in which human and non-human agents are present. Several of these systems are involved in medical context, for clinical protocols and follow-up processes, or in general in the rehabilitation field, for instance to restore the motor functionality of limbs. Since great improvements have been performed in recent years, social robots became more similar to human entities; they allowed us not only to connect people around the world with telepresence systems, but in some cases, they even acted as family members, caregivers, and nurses in the companionship of people with dementia, autism spectrum disorder, and other mental or physical disorders. Some of the greatest advantages provided by social robots applied in older adult-care consist of increasing individual independence, facilitating their everyday routine, limiting human caregivers intervention, and making easier communication with family members using remote connections [1]. Furthermore, robotic animal companions have been developed and adopted inside care facilities to carry out pet therapy provided to the patients. In other cases, social robots can be used to reduce children’s pain and anxiety related to surgery, adapting their behavior to the estimated emotional state of children, which has reduced their capability to communicate their possible discomfort [2]. Moreover, ethical implications of social robots and embedded artificial intelligences have to be carefully considered in designing their behavior, especially when they are involved in pediatric environments [3]. Although this research field is rapidly progressing and even more effective solutions are being employed, numerous issues and social stigma still have to be overcome. Thus, multidisciplinary research teams (i.e., physicians, engineers, and social scientists) have to be involved to identify social robots design and way to behave, which can reduce people’s negative attitude toward them [4]. The literature proposes different scientific reviews of social robots, mainly focused on more or less broad application sectors; nonetheless, there is an absence of scientific reviews that address the functional aspects and technical features used to achieve those functionalities. For this reason, this work focuses on the state of the art of social robotics from a technical perspective, specifically in medical, healthcare, and educational environments

According to a design-centered approach proposed by Bartneck and Forlizzi [5], a social robot is an autonomous or semi-autonomous robot that interacts with humans by following the behavioral norms expected by the people with whom the robot is intended to interact. Their definition presupposes three conditions: the robot has to be autonomous; depending on the case it has to interact cooperatively or non-cooperatively; and it has to recognize human values and roles. Furthermore, Bartneck and Forlizzi asserted that verbal and non-verbal communication between social robots and the human entities is fundamental. These systems must be perfectly matched with their final user: a lot of work must be accomplished in order to obtain an appearance that avoids the “Uncanny valley phenomenon”. In fact, in 1970 Masahiro Mori hypothesized that once the robot has reached a threshold value of lifelikeness, it may evoke a negative response in the people. This phenomenon is strongly related to the appearance of robots and its impact on human targets. The effectiveness in the establishment of a relationship that provides benefits for users is defined by a range of robots’ similarity to the human being in terms of behavior and aesthetic features. Thus, the design of humanoid robots may be limited, and the development of more machine-like systems may be preferred to more realistic one. Emotion and expression recognition is also important to generate a strong connection between humans and cyber systems. Moreover, it is essential to design a robot which transmits trust and is able to behave as much as possible in the most natural way. For current robots, all these features can be achieved thanks to several sensors, actuators, and devices connected to complex hardware and managed by operating systems where programs run to guide robots in the environment, allowing them to relate and be part of the daily lives of humans. In this context, this paper aims to provide a mapping of methodologies and various technical solutions required to build a social robot for healthcare environments. The review is performed analyzing data extracted from three specific fields: SL (the scientific literature, investigated through the Scopus database (Elsevier ©)), PL (the patent literature, analyzed querying the Espacenet database by the European Patent Office EPO), and MA (a wider market analysis, performed thanks to the Google search engine (Google LLC, Mountain View, CA, USA)). This work is organized according to these three branches of investigation: the first section considers the scientific research; the second section collects patents data; and the last part depicts the heuristic research on the market solutions. For each section, the data selection procedure and performed data analyses are described in a first Materials and Methods subsections; then, the results are presented and discussed to capture, explain, and reorganize the most relevant technical characteristics of social robots for healthcare environments. In conclusion, this paper consists of a very extensive, technical review, that also focus attention on ethics and privacy concerns related to the deployment of social robots in healthcare environment, although not being a primary goal. Summarizing the principal features of this work, the following are presented:

- It stands out as a broad and comprehensive review, both from the temporal and sectorial points of views (i.e., years, scientific field, patents field, and global market);
- The healthcare environment is very wide and diverse; thus, specific applications, stakeholders, and diseases in which SRs are involved are presented;
- Technical devices and requirements of SRs are discussed in detail;
- Guidelines for a multidisciplinary team of researchers involved in social robotics field are reported. This leads to the development of a cyber solution capable of establishing effective human–robot interactions.

### Organization

In this paragraph, the general organization of the present work is reported. The complexity and amplitude of the field under investigation have required an adequate structure, capable of providing all the information, in an effective and easy understandable manner, to whoever is interested in social robotics applied in a healthcare environment. The structure of this review can be summarized using the compact framework described below:

- Scientific literature research and an accurate analysis of the Scopus database (Elsevier @) are reported. In this first part, the method used to obtain data is presented, and according to different parameters information is elaborated upon, mapped, and filtered to present results in the Discussion (Paragraph 5).
- Patent research (in this case, the database involved is the Espacenet database) and information on analytical methods used and data treatments are presented. In this second part, a classification based on the international patent classification (IPC) has highlighted important patents reported and analyzed in the Discussion.
- Market research has been executed through Google search engine (Google LLC, Mountain View, CA, USA), and several pieces of information have been reported about market goals, limitations, and global dimension. Also in this case, the obtained results have been properly discussed in Paragraph 5.

Information, technical data, and descriptions of solutions coming from the three sources mentioned above have been obtained using the same taxonomy (i.e., analytical review and maturity review), and then, they have been incorporated and discussed extensively in the Discussion part. The present work is intended as the optimal starting point for multidisciplinary teams of scientists that have started studying social robots, and their final aim may be developing their own project. This review provides information about the most active countries and institutions in the sector, the rapid growth of interest around this field, both from the industrial and scientific communities point of view, and the technical innovations that have occurred over time. 

## 2. Scientific Literature Research

### 2.1. Materials and Methods

#### 2.1.1. Data Selection Procedure

**Figure 1:**
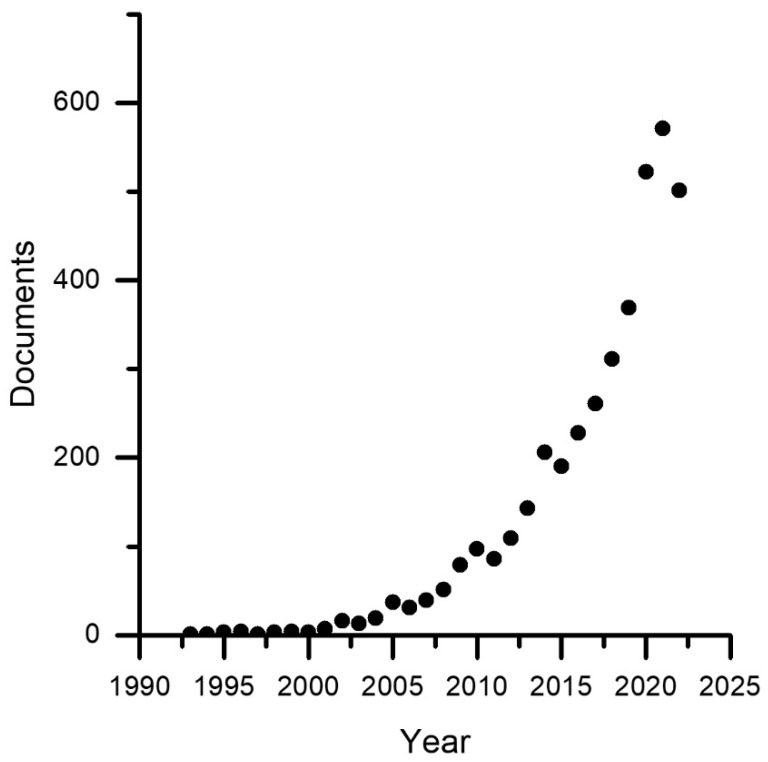
Number of documents related to the year of publication in the range 1992–2022.

**Figure 2:**
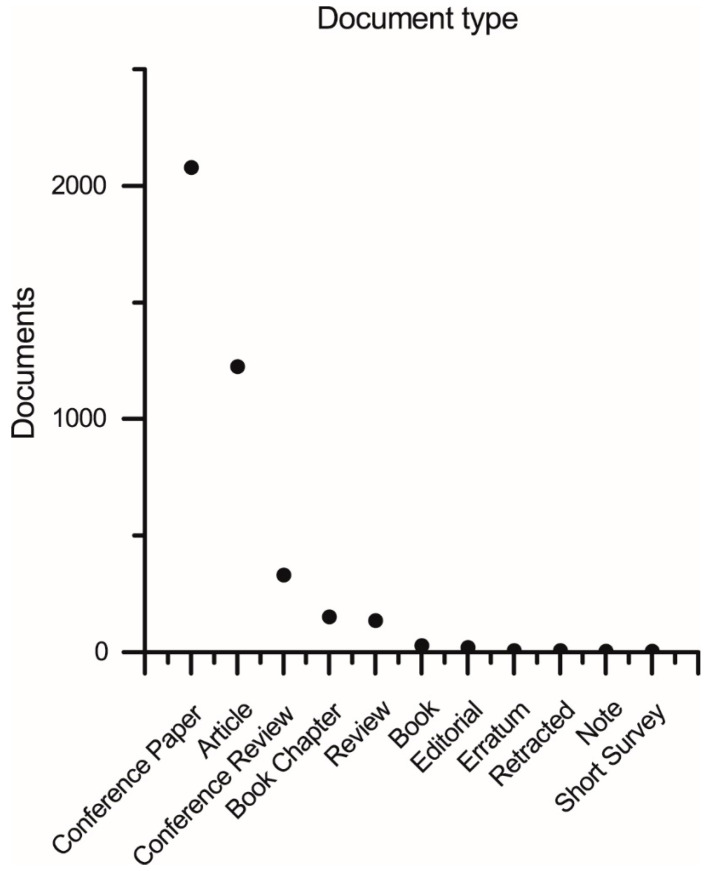
Number of documents related to the type of publication.

The literature research was performed on documents indexed in the Scopus database (Elsevier @. Conceptual keywords for the query were social and robots, combined with medical or healthcare to focus the review. For the purpose of the current analysis, those keywords or variations are expected in the title, abstract, or keyword metadata field of the documents. According to these considerations, the following search string was combined: “TITLE-ABS-KEY (social AND robot* AND (*medic* OR *care OR clinic* OR therap* OR treatmen* OR disabilit* OR dementia OR autism OR diabet* OR stroke OR pain))”. This query, run for the last time on the 13 March 2023, provided 6036 research products. Results were then filtered applying as inclusion criteria the classification of the document in at least one of the following subject areas of the Scopus database: Computer Science (“COMP”) and Engineering (ENGI). The subset of products compliant with the inclusion criteria was composed of 3977 documents, distributed among years and by document type as Figure 1 and Figure 2 describe, respectively. Thus, the collected results have been stratified in different ways to stress, respectively, temporal, topic, and geographical correlations with publications by year, documents by subject area, documents by country, and documents by source type. This analysis has been performed directly with Scopus Elsevier instruments.

In Figure 1, only the 1992–2022 range is shown for greater comparability between years, although it should be noted that indexing in one year is generally completed by June of the following year, so a careful analysis shows a very high and continuous growth trend in scientific production since at least the year 2003. From Figure 2, we can infer some observations that will be further elaborated in the section on the results discussion. We observe a notably greater number of scientific conference products than journal products. This phenomenon is typical of disciplines with a high rate of innovation, such as computer science, where the lengthy publication times typical of academic journals are incompatible with the innovation rate. It is evident that the journal plays an important role in establishing scientific results and laying a solid foundation for future development; therefore, researchers also utilize this publication venue. Journals are recommended for obtaining reliable information, but conference materials cannot be ignored. This outcome is also a consequence of the fact that the current scientific work focuses on the technical aspects of social robotics. The high number of scientific reviews in both journals and conferences, comparable to medical disciplines, is an additional intriguing phenomenon. In medical disciplines, the phenomenon is a result of meta-analyses that combine difficult-to-replicate experiments involving human subjects. In the field of social robotics, however, the authors believe that this phenomenon is due to the high level of interdisciplinarity, which will be explored in greater detail in the discussion section, and the inability of a single reader to delve into all the disciplines required to comprehend and develop a social robot. For this reason, researchers in the field of social robotics feel the need to write and read scientific reviews, which represent a moment of interdisciplinary synthesis and are a crucial tool for fostering team cohesion.

**Figure 3:**
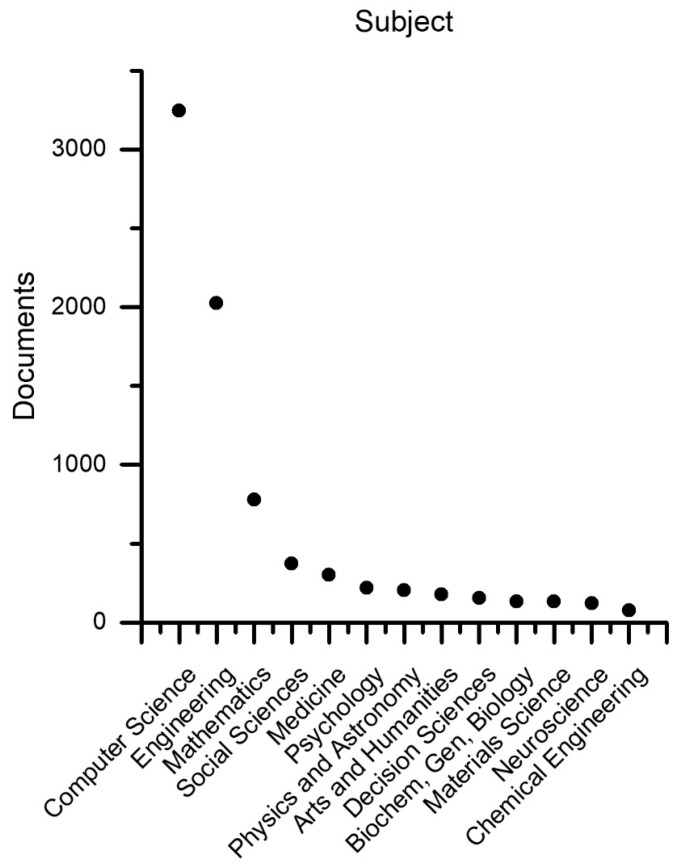
Number of documents by subject area.

**Figure 4:**
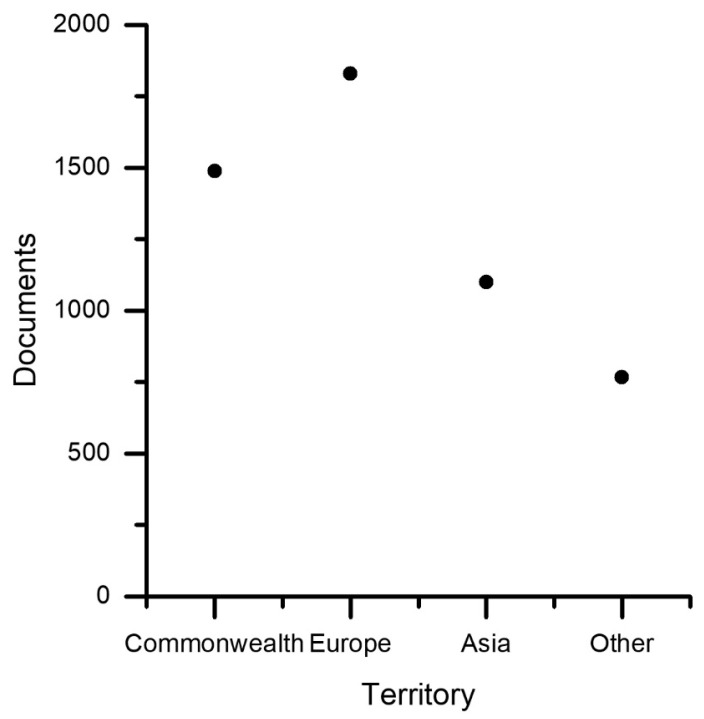
Number of documents related to the affiliation territory.

Scientific products segmented by scientific subject areas are collected in Figure 3. It should be specified that a document can belong to multiple subjects simultaneously, which represent, therefore, labels attributed to the individual document. Scientific subjects are associated with subject areas; in fact, often and traditionally, editorial sources deal with disciplines and not applications, apart from special cases. For this reason, Figure 3 can be interpreted to understand which disciplines are most involved in social robotics for technical design purposes (because of the particular selection string). As might be expected, computer science and engineering are determinants, but mathematics (it would be better to specify logic, theoretical computer science, and control theory), social science, medicine, and psychology are relevant. Physics and astronomy are very general fields, so it is not appropriate to consider them. Arts and humanities, as well as decision sciences, are interesting to emphasize in order to accurately represent the human dimension and develop decision strategies in complex situations. Chemical, biological, and materials sciences are absolutely relevant for proposing innovative materials that mimic biological ones but also for studying material interactions with humans. Neurosciences are present, although in limited quantity, because the Scopus classification includes in this subject only neurology and related disciplines, which represent a very broad field in general and have great scientific impact but are limitedly involved in social robotics compared to, for example, cognitive sciences, which Scopus tends to include in the social sciences. The spatial stratification (Figure 4) is much more complex than it appears in the breakdown by countries. In fact, different nations have different geographic sizes, numbers of inhabitants, and numbers of active researchers and are able to be composed of various states. A compromise was chosen by identifying three determining geographic areas: the Commonwealth, Europe (including the European Union, Eastern Europe, Russia, and Israel), and Asia, as well as including the rest of the world in the “other” container. Certainly, this segmentation has many weaknesses; for example, it is difficult to compare the life expectancy and gross domestic product of former USSR areas with those of Western European areas. On the other hand, Japan has great similarities, e.g., with Italy, due to the presence of a strong drive for tradition side by side with a great tendency for innovation. However, from the proposed segmentation and a more in-depth analysis, which is omitted here, it appears that areas where the following factors are present are facilitated in the production of research in the field of social robotics: good social sensitivity combined with respect for the individual, high life expectancy combined with a good gross domestic product per capita, and, above all, a holistic cultural dimension.

#### 2.1.2. Subset Pre-Filtering

In order to obtain a reduced dataset, focused on documents which have technical relevance in the social robotics field, the subset mentioned above has been treated analyzing the title and the abstract of each document. This process has been carried out assigning a numerical marker (0, 1, 2) to each element of the subset imported in an Excel worksheet, trying to answer to three research questions: (0) Can the described system be used as a social robot? (1) How is it used, or what is needed to make it a good social robot? and (2) What is the system composed of? The marker “0” has been therefore related to documents that describe the result of social robots’ involvement in medical trials or any cyber-apparatus, such as exoskeletons or surgical robots, incapable of acting as social partner with the human being, even if characterized by a high level of autonomy. Investigations on users’ opinion and ethical and social impact of social robots on people have been grouped in this category as well. The marker “1” has been assigned to documents that provide information about the development and fields of application of social robotic platforms and prototypes but lack technical information about embedded equipment reported. The main focus of the documents is evaluating the medical path in which the systems have been inserted and their effectiveness, but more technical information can be obtained through analyzing the references. The marker “2” has been related to documents that contain technical information about social robots’ equipment and detailed descriptions of robotic platforms. Moreover, technological improvements of already known robotic features (e.g., algorithms, frameworks, sensors, and motors) are grouped in this directory. In conclusion, 77 documents (marked by value “2”) passed the selection process and have been assembled in the final reduced dataset for further analysis.

#### 2.1.3. Data Treatment

The subset and the reduced dataset are separately analyzed. For the first one, a bibliometric analysis has been performed mapping the whole literature with “VOSviewer” (Laiden University, The Netherlands), to highlight keywords and authors bibliometric networks, respectively. 

##### Bibliometric Analysis

**Figure 5:**
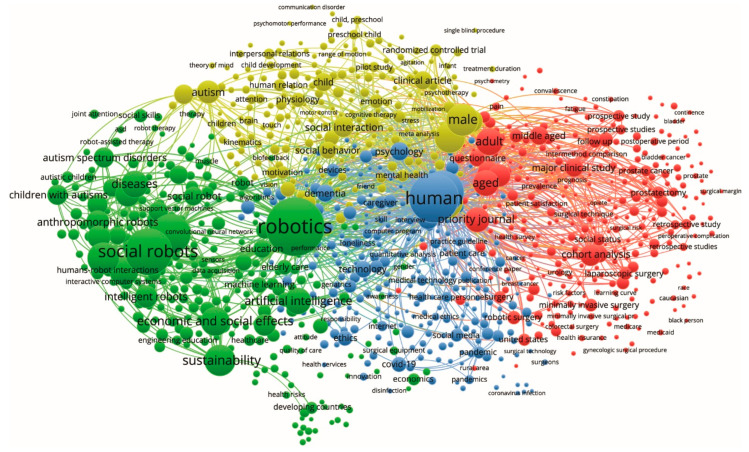
Keywords cloud obtained from VOSviewer software has been reported.

Results in the subset are firstly presented with a map approach [6] to enhance the bibliometric network, in terms of keywords using the software VOSviewer. Figure 5 shows a keywords network in the “network visualization” display mode by selecting “Co-occurrence” and “Authors-Keywords” to extract the map. Figure 5 identifies the structure of the research area of social robotics and drives eventual heuristic sub-searches, representing the most relevant keywords as wider and interconnected poles.

##### Taxonomy

The reduced dataset of 77 documents has been investigated with respect to two different analysis processes, enabling the synthesis of an analytical and maturity review.

##### Analytical Review

In this phase, in order to carry out the reduced dataset analysis, the following classification has been used:

- Embodiments: This category focuses on describing the main structure of the robots related to their specific functions and field of application;
- Appearance: This category considers the appearance of social robots applied in the healthcare area, discussing on materials, shapes, and innovative techniques used to produce some aesthetic parts of the robots;
- Movements: In this category, the motion capability of robots has been analyzed, concerning the degrees of freedom and actuators used to generate movements;
- Sensors: In this category, the sensory equipment of social robots has been described. It has been classified with respect to the measured target magnitude and main aim of each type of device;
- Algorithms: This category gathers algorithms used to define the system interactions between environment and social partners;
- Hardware and Connectivity: This category describes the involved communication protocols and the adopted hardware and architecture;
- Human–robot interaction: This category describes methodologies and devices that allow to share information between social robots and human being and controlling systems used to direct robots’ assistance.
- Ethical, privacy, and security issues: This category describes aspects that lawmakers are focusing on most, because of several potentially controversial implications.

##### Maturity Review

In this phase, the technology readiness level (TRL) scale defined by the European commission [7] has been used to carry out the maturity analysis of solutions described in the reduced dataset. This scale has been used to determine the development state, maturity, or market uptake of the system under investigation. It is characterized by the 9 levels reported in Table 1. Adaptations to medical environment have been considered during the analysis process. 

The used evaluation criteria have required the analysis of the full texts, considering mainly the environment of experimental trials performed, the procedure of validation, results reported, and concluding remarks.

### 2.2. Results

#### 2.2.1. Results for Subset Sub-Filtering Process

The subset has been stressed according to the above-described taxonomy. The numerical markers (0, 1, 2) have been used as labels to identify which documents have passed the validation process and which one have been excluded:

- Marked by value “2”, 96 documents have passed the validation process, and they have been subjected to deeper analyses (Analytical review and Maturity review);
- Marked by value “1”, 1021 documents have not passed the validation process, and a further review is required;
- Marked by value “0”, 2860 documents have not passed the validation process.

**Figure 6:**
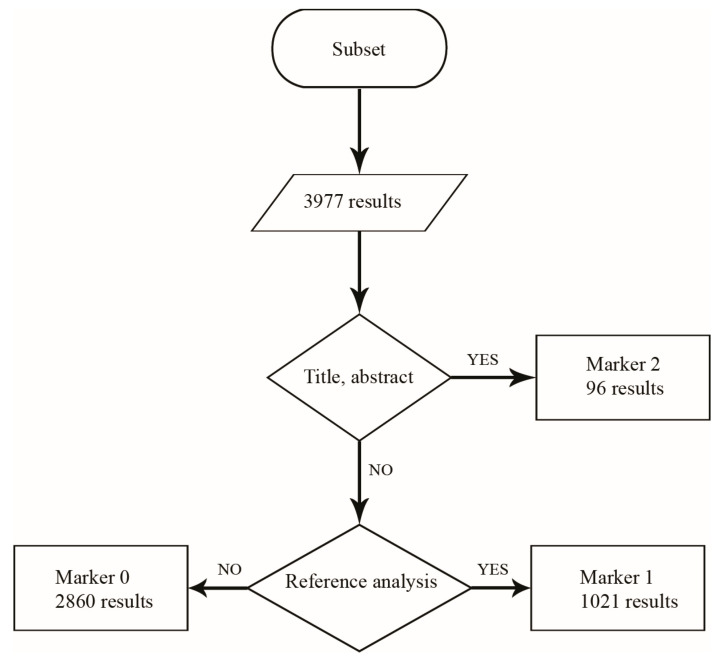
Flowchart diagram of the research: the starting subset has been obtained from Scopus database after the application of the inclusion criteria; tag 2 identifies very relevant documents; tag 1 labels documents with some relevant information; tag 0 collects not pertinent documents.

In conclusion, documents labeled with tags “0” and “1” have been excluded from the present review, and the result of the sub-filtering process is reported in Figure 6.

It is important to specify that the articles that were included, in addition to being relevant in terms of content related to social robotics for health, present technical information about how a specific robot or some parts of it are constituted. This scientific review therefore focuses on technical aspects of social robotics, which, as we will see in the discussion section, cannot be completely separated from other aspects because social robotics is inherently multidisciplinary, and application in the health sector adds additional areas of scientific expertise.

#### 2.2.2. Bibliometric Results

The set of keywords appearing in each document, both author-defined and indexed, was represented using VOSviewer to identify a bibliometric co-occurrence map, i.e., the relatedness of the items is determined based on the number of documents in which they occur together. A full counting method was adopted; i.e., each co-occurrence has the same weight in the analysis. To be represented, a keyword must appear with a minimum number of occurrences of five.

Two classes of observations emerge from the analysis of the keywords depicted in Figure 5. First, it is possible to identify the most recurring keywords in the class of articles considered, namely robotics, human, and social robots, alongside which emerge some keywords that could have been imagined, such as diseases, aged, adult, autism, artificial intelligence, and some less obvious ones, such as economic and social effects, sustainability, male, priority journal, and cohort analysis. Focusing on the less obvious keywords, economic and social effects, as well as sustainability draws attention to the strong economic and social motivations underlying the investment in social robotics for health. Interesting is the appearance of the keyword male, which alludes to the aspect of gender that needs to be explored, also because robots have no inherent gender and their entry into a society of gendered individuals represents an element of complexity. Still, the term priority journal needs to be explained because it can have a multiplicity of meanings, in this case, generally representing the set of priorities of the 2030 Agenda for Sustainable Development Goals. The presence of the term cohort analysis, on the other hand, alludes to the fact that even technical studies of social robotics can be validated through experimental interaction approaches on human subjects using methods typical of medical disciplines. A second class of observations can be made on the macroclasses of keywords formed by proximity that do not exactly coincide with the segmentation into colors assigned automatically by the software in Figure 5. One notices keywords typical of technical disciplines on the left and keywords typical of medical disciplines on the right, but in the middle of the picture, keywords typical of the social sciences and a kaleidoscope of other disciplines dominate, holding together technology and medicine through a complex web of cultural glue.

#### 2.2.3. Analytical Results

**Figure 7:**
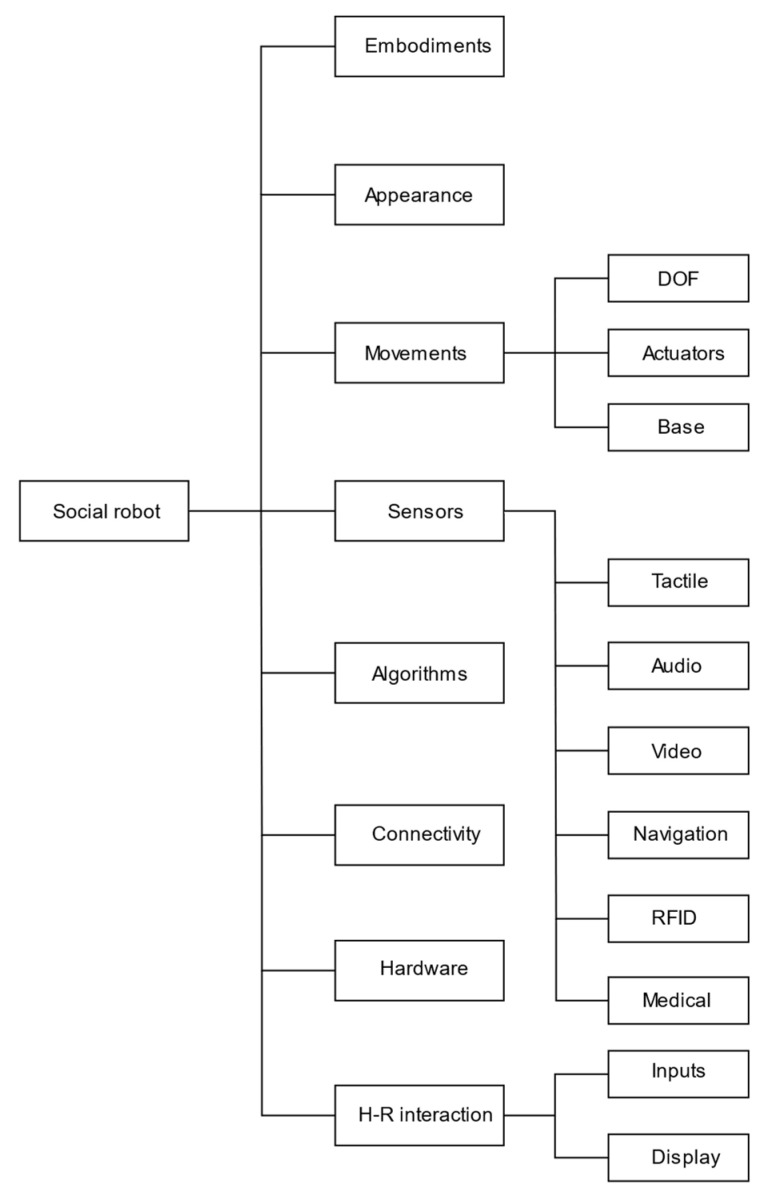
Blocks diagrams of the analytical analysis’ general arrangement.

**Figure 8:**
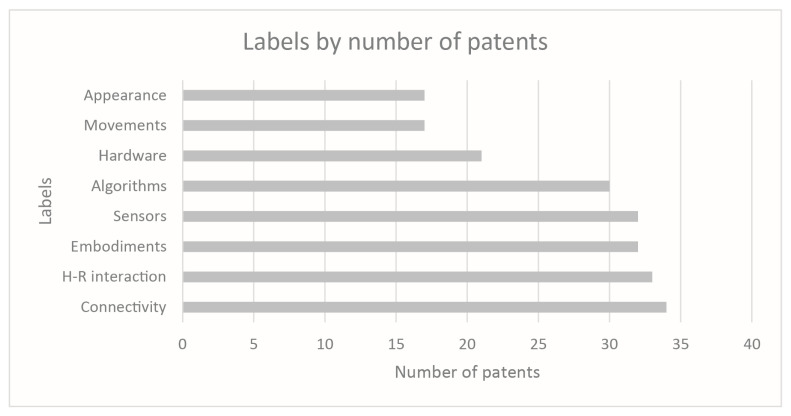
Number of documents that contain detailed description according to the related labels.

The analytical review has led to the topic arrangement reported in Figure 7. A second classification layer has been identified for the following blocks: “Movements”, “Sensors”, and “Human-robot interaction”. It has been used to direct and focus the discussion phase on specific targets of interest. The results in Figure 8 show which topics are more addressed by the documents in the reduced dataset. The indexed labels reflect a homogeneous treatment of any type of technical equipment and particular attention is paid to “Sensors” and “Algorithms” fields.

#### 2.2.4. Maturity Results

**Figure 9:**
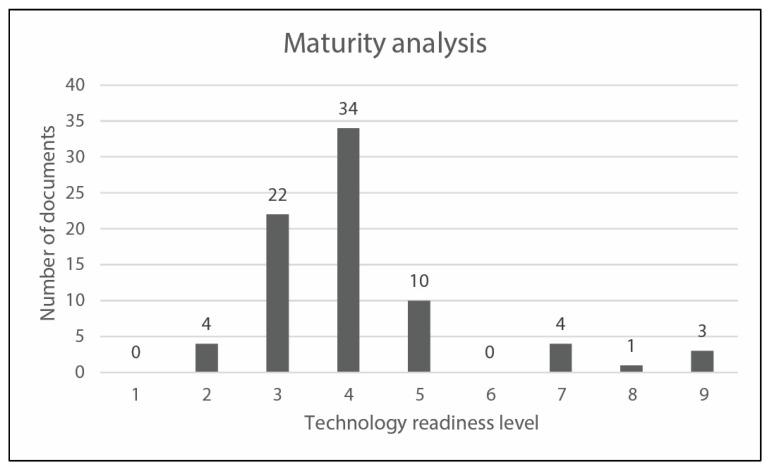
TRL (Technological readiness level) for every document, assigning a numerical label from 1 to 9 in relation to its maturity state.

The reduced dataset has been treated investigating the “Technology Readiness Level” (TRL) of the described solutions to analyze the status of development of research project. This type of evaluation, reported in Figure 9, can be a useful parameter to investigate and discuss the implementation of social robots in real-cases and field trials. Moreover, the TRL analysis can fill the gap between SL analysis, PL analysis, and MA analysis.

## 3. Patents

### 3.1. Materials and Methods

The patent research was conducted on the Espacenet database. The research was performed for the last time on the 3rd of January 2022, considering patents published between 1995 and 2021. An ADVANCED SEARCH based on “all text fields or names” was conducted using the string “(Social) AND (Robot) AND (Healthcare)”, that produced 1985 results, collected in the main dataset. This is composed of all the available results, since at this step of the process no exclusion criteria have been applied to select a specific patent inside the patent families. In order to identify the application information of every patent in the main dataset, focusing a subsequent analysis on a narrower and more specific collection, the international patent classification (IPC) index has been considered. The IPC is a classification system that organizes inventions and their documents into technical fields that cover all areas of technology. The IPC has a hierarchical structure and is subdivided into sections, classes, subclasses, groups, and subgroups [1]. The analysis of the main dataset has been based on three specific IPCs individuated by a group of experts:

- B25, hand tools; portable power-driven tools; manipulators [1];
- A61, medical or veterinary science; hygiene [1];
- G06, computing; calculating; counting [1].

Thus, the main dataset has been treated, and 512 patents have been excluded by further analysis processes. Once the first selection criterion has been applied, 1457 patents belonging at least to one of the previously reported IPC groups have been identified. These results have been arranged in the filtered dataset, and to perform a more detailed analysis, title and abstract have been considered to classify them as follows:

- Marked “III” patents: These documents are related to robots involved in social and/or crowded environments for personal assistance, medical, and telepresence purposes.
- Marked “II” patents: These documents are related to non-specific embodiments and general technologies that are not yet implemented in social robotics field, or their aim is not expressly referred to medical purposes and/or robotic applications.
- Marked “I” patents: These documents include systems that are not related to social robots and their application in medical and assistance fields. For instance, patents focused on industrial devices or methods and products used to treat infections and diseases have been marked with “0 tag”.

**Figure 10:**
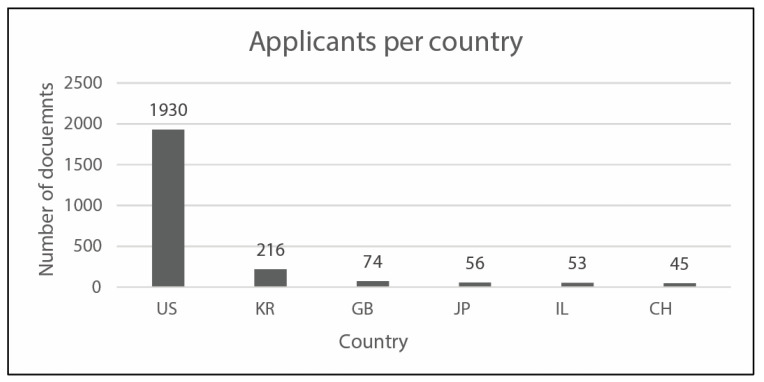
Applicants per country chart. Country is the country or organization where the patent application was filed or granted [1].

**Figure 11:**
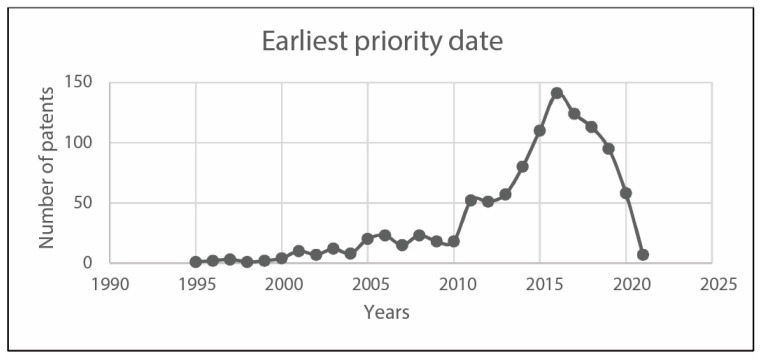
Earliest priority date chart. Earliest priority date is the filing date of the very first patent application for a specific invention. Within 12 months of that first filing, a subsequent patent application for the same invention can be filed claiming this “priority right” [1].

**Figure 12:**
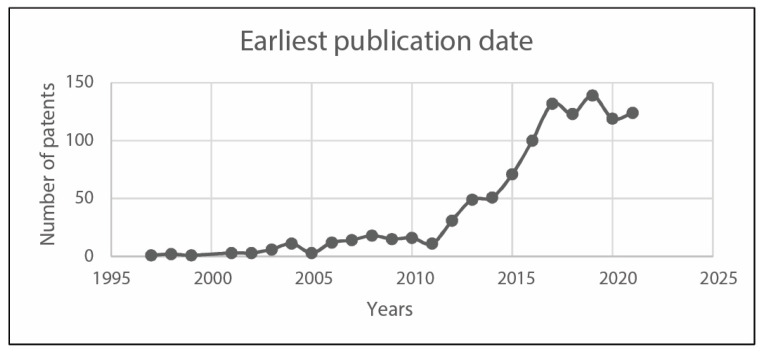
Earliest publication date chart. Earliest publication date is the date on which a patent application is first published. It is the date on which the document is made available to the public, thereby making it part of the state of the art [1].

**Figure 13:**
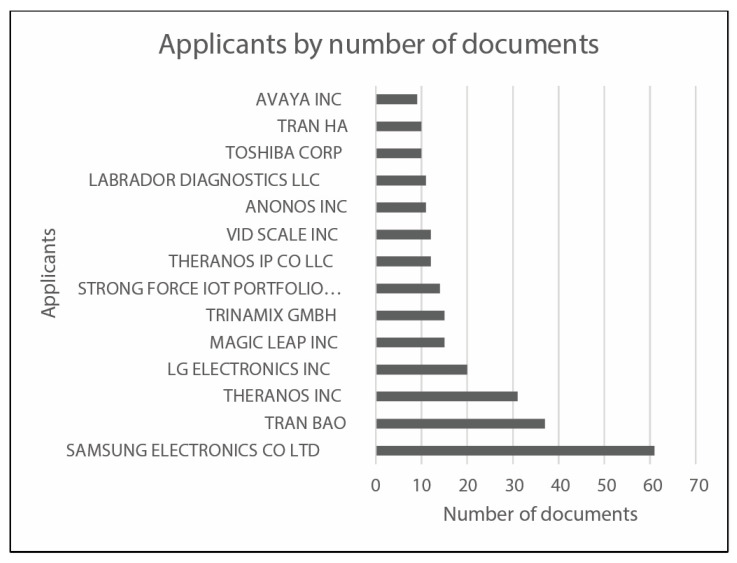
Applicants by number of documents chart. Applicant is a person (i.e., natural person) or organization (i.e., legal entity) that has filed a patent application. There may be more than one applicant per application. The applicant may (but need not) also be the inventor [1].

In the following, Figure 10 reports the applicants per country, whereas Figure 11 and Figure 12 analyze earliest priority and publication dates, respectively. Figure 13 finally collects the applicants by number of documents. These figures allow describing at a glance the field under investigations, in terms of temporal, geographical, and industrial coordinates. Moreover, further research may be differently oriented to develop more specific strings for Espacenet search engine, involving a narrow group of countries or applicants.

### 3.2. Results

**Figure 14:**
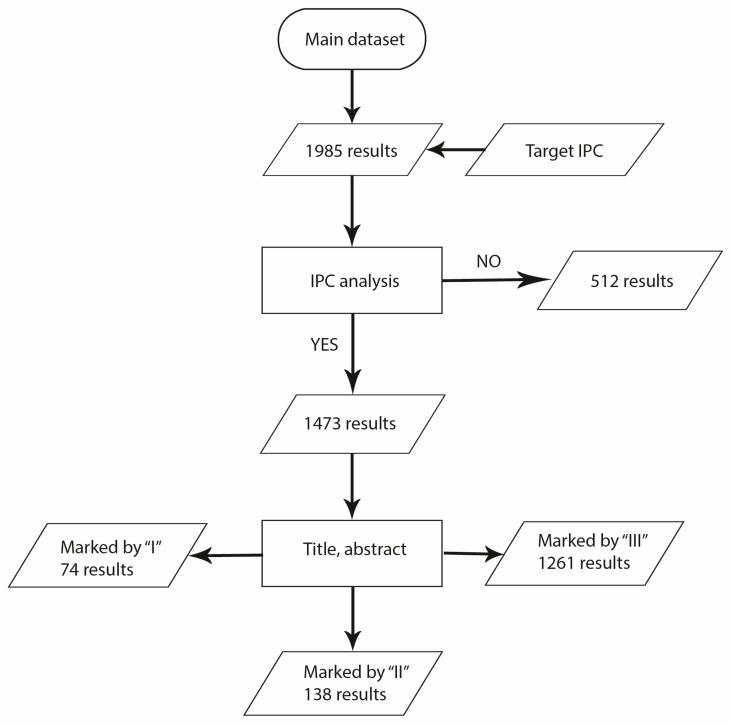
Flowchart of patent analysis.

The main dataset of 1985 results obtained by the Espacenet database has been treated as mentioned in the previous section. Figure 14 shows the flowchart of the applied data selection process. 

**Figure 15:**
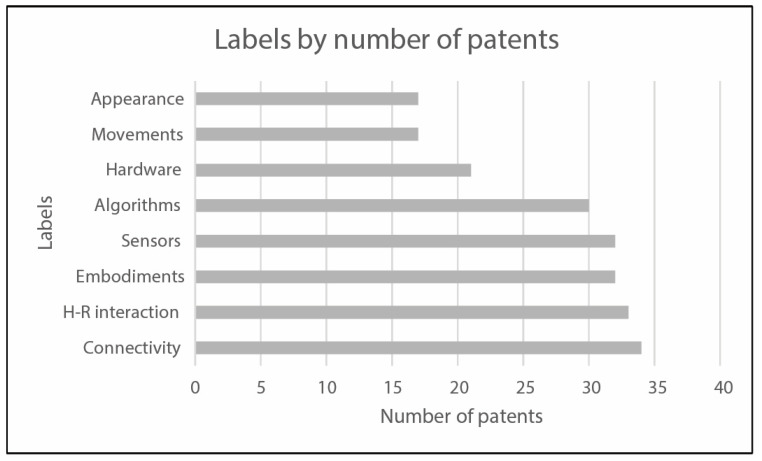
Number of patents that contain detailed description according to the related labels.

The final set of 74 collected results has been stratified according to the same classification labels used in the scientific literature research and reported in the previous figure, Figure 6. For each patent family, the analytical analysis of the final dataset has been carried out on the patents presenting the most recent publication date; thus, the resulting treated documents are 42. In order to determine which areas are most assessed by the collected patents, the frequency distribution of documents among identified labels has been investigated: Figure 15 presents the most frequently addressed labels for patent classification and the corresponding number of patents that refer to the specific label.

**Figure 16:**
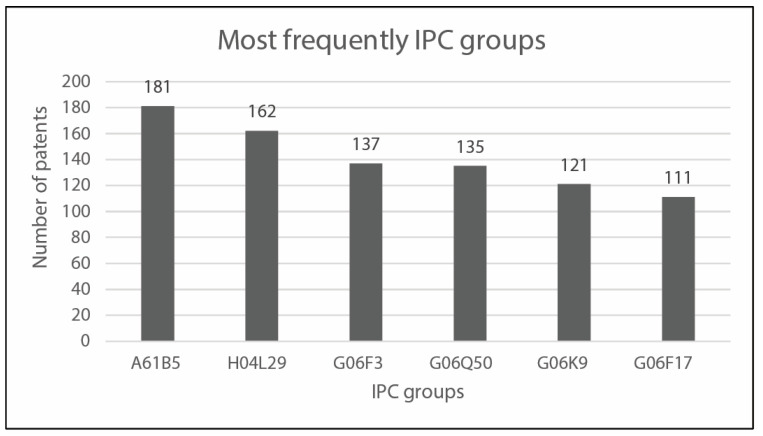
Main IPC groups. Descriptions from Espacenet website are reported in the following [1]: A61B5: Measuring for diagnostic purposes; Identification of persons; H04L29: Arrangements, apparatus, circuits, or systems, not covered by a single one of groups; G06F3: Input arrangements for transferring data to be processed into a form capable of being handled by the computer; Output arrangements for transferring data from processing unit to output unit, e.g., interface arrangements; G06Q50: Systems or methods specially adapted for specific business sectors, e.g., utilities or tourism; G06K9: Methods or arrangements for recognizing patterns; G06F17: Digital computing or data processing equipment or methods, specially adapted for specific functions.

Figure 16 reports the IPC groups most frequently present in the main dataset, whereas Table 2 allows correlating every IPC group with its frequency of occurrence.

**Figure 17:**
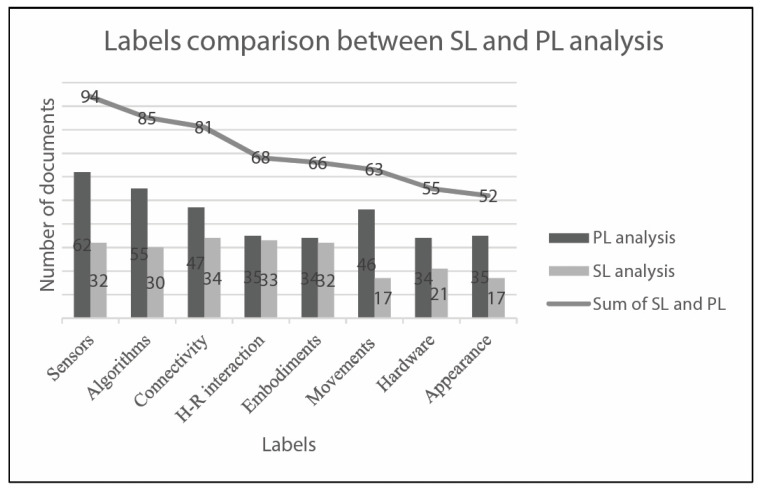
Labels comparison between SL and PL analysis of the reduced dataset (SL) and final dataset (PL) with the addition of the cumulated to identify the review’s amplitude.

Figure 17 shows a comparison between SL and PL analyses in terms of indexed labels and their overall collection. It must be considered that the SL dataset (77 items) and PL dataset (42 items) have different dimensions. The overall collection made by the sum of indexed labels of scientific and patent datasets aims to identify the dimension of the literature investigated in the following Section 5 “Discussion”.

## 4. Market

### 4.1. Materials and Methods

The market analysis has been carried out between June and August 2021 querying the Google search engine. This heuristic research has been focused on commercialized products and the in-real application of findings related to scientific and patent filtered literatures. Thus, in this case random queries based on “Social”, “Robots”, and “Healthcare” keywords have been run. In order to obtain detailed information on the social robotics market and its dimension, not even documents that consider economic, social, and political aspects have been a priori excluded in the collection of final results. The use of a single search engine constitutes the main limitation of this research process; possible improvements may be related to the choice of other web search engines as additional data sources. Finally, the same taxonomy adopted in the analytical analysis of the scientific literature for the study of the reduced dataset has been used to treat the found products.

### 4.2. Results

The integration of data collected by the market analysis has been preceded by considerations about market dimensions, goals, and applications of social robots in specifical medical and assistive tasks. Concerning service robots, i.e., those social robots specifically able to assist humans in performing useful tasks, the analysis of their sales by industry in 2014 shows that robots were more involved in defense, farming, and logistics than in the medical field [8]. However, in the last decade, the aging of the population has increased the need of support systems for medical personnel. In fact, it is expected that the social robots’ market capitalization will annually grow of 12.68% starting from the USD 395.577 million in 2019 [8]. This amount is related to several devices which have already been deployed in hospitals for surgical purposes and physical therapy (e.g., exoskeletons), but many other fields will be improved by robotics in the near future. For example, SARs (socially assistive robots) will be very useful in taking care of people affected by dementia or neurodegenerative diseases. Several companion bots such as PARO [9] and AIBO [10] have already been implemented in care houses or private homes, and their efficacy has been confirmed. Thus, they consist both of optimal currently used solutions and a starting point for the economic growth of this sector. As suggested by the scientific analysis on the Scopus database, North America and Pacific Asia will benefit from their research program in social robotics, and they will act as a protagonist in the global market by the end of 2026. For instance, in 2018 the Japan government allocated USD 100 million investment to develop and deploy nursing robots. Also, South Korea is currently employing great resources for social robots in healthcare. In fact, according to the World Bank’s estimations, the aging population in South Korea is rapidly increasing, surpassing the Japanese one and reaching 37% in 2045. Another cue to enlarge investments has been provided in this last two years by COVID-19 pandemic, in which robots have guaranteed both caring of people and social distancing. In this specific situation, exploiting a medical robots classification proposed by Khadidos (i.e., disinfecting/spraying robots, robotic hospitality, telepresence robot and surgical robots, and robotic hospitality) and based on their main function, it can be observed that devices involved in robotic hospitality (i) and telepresence solutions (ii) are the most propelling considering the near future medical robot market. Considering the two above mentioned categories, robotic technical endowment must guarantee:

(i) Food and medication distribution (e.g., Sona-2.5, Zafi medic robot, and KARMI-Bot, CO-Bot) through a high degree of automatic handling (e.g., SLAM algorithm) and load-carrying capacity (above 15 kg);
(ii) A high level of robot interaction capacity with users or patients. Touchscreens, displays, and cameras constitute essential devices to provide teleconference ability to the robots (based on Web real-time communication), while algorithms such as speech recognition, emotional state recognition (e.g., monitoring systems based on deep neural network), and SLAM localization allow robots to collect data on people and the environment [11].

## 5. Discussion

Social robots are becoming increasingly significant in healthcare, and technical advancements will increase their usage and impact. Patients’ health may be affected by these robots’ emotional support. These systems may improve healthcare accessibility, efficiency, and cost. One of its biggest advantages in healthcare is emotional support. Social robots may reduce anxiety, depression, and other mental illnesses, according to research. Social robots have treated several disorders in psychiatry, pediatrics, geriatrics, and rehabilitation. Interactive play and behavioral feedback from social robots have helped autistic patients improve their speech and social abilities. In the case of dementia, social robots have been utilized to provide cognitive stimulation and companionship to patients through talking, playing games, and sending daily reminders. In the rehabilitation phase, stroke patients have utilized this technology to provide tailored feedback, assistance with exercises, and motivation for recovery. In addition, social robots have been utilized to provide therapy, tailored support, and advice for various mental illnesses, such as depression and anxiety, as just mentioned, supporting patients through behavioral activation approaches. Social robots are being employed in the healthcare sector to improve patient communication and education. For instance, a social robot may be utilized to educate patients on their medical issues, prescriptions, and therapies. This can aid individuals in comprehending their healthcare requirements and improve their overall health. Social robots allow healthcare practitioners to offer distant care, lowering costs and increasing access. This technology can monitor patients at home, eliminating the need for hospitalization and frequent doctor visits. Also, this has freed up healthcare resources for those with the highest requirement. The impact of social robots on healthcare personnel is a second effect. Social robots can alleviate the workload of healthcare professionals by performing regular tasks such as collecting vital signs and patient data. This permits physicians and therapists to concentrate on more difficult duties and spend more time with patients. In addition to these effects, social robots have contributed to an increase in patient satisfaction with healthcare experiences. Social robots made patients feel more comfortable and involved, improving their treatment experience and increasing their probability to seek further care. Thus, it might enhance patient outcomes, save healthcare costs, and boost treatment availability. 

### 5.1. Embodiments

Social robots are involved in various fields, and their features are extremely related to the function that have to perform. Thus, functionality and structural and aesthetic characteristics must be completely synchronized, and different types of social robot, such as the one involved in entertainment, companionship, monitoring, delivering supplies, or rehabilitation, are subjected to different engineering processes (i.e., conception, designing, and manufacturing) [12]. Social robots can be preferred in several different embodiments depending on the target people, environment of work, and the tasks to carry out:

- Humanoid robots are systems characterized by human-like appearance. They can present a virtual or a physical face; the first is shown on a screen or by LED arrays where the mouth, eyes, and nose can be displayed; the second can be realized by three-dimensional printing or other processes and be coated by synthetic skin or left uncoated. Moreover, they can present legs and/or arms to improve their capability of interaction.
- Pet companion-bots are systems designed to replicate the shape of a companion animal such as cats, dogs, seals, etc. The most famous embodiments are AIBO and PARO. They are used for pet therapy, when using real animals could be difficult or impossible due to allergies or when the patient is not able to take care of the companion.
- Telepresence robots are a system coupled with wheels and a motor drive unit. They have a vertical structure which ends with a display or a touchscreen, capable of showing videocalls or a virtual human face to better interact with people.

This latter robotic field in particular is rapidly progressing and benefits other relevant technologies [13,14,15]. A classification of different robots according to their embodiments is listed in Table 3.

The patent state of the art resulted usually in generalized embodiments with unrecognizable animal or human traits, because these innovations are applicable to a wide range of robots with different embodiments. Different patents have defined the main characteristics and the principles of operation, the customization of which according to the final users’ needs in specific environments will be established subsequently [58,59,60,61,62,63,64,65,66,67,68]. Products currently available on the market exhibit several types of embodiments intended for different application sectors:

- Small-sized robots are desktop-designed bots, a very common solution inside the market. They are involved in children’s educational environment and personal assistance (i.e., Dinsow Mini [69], Little Sophia [70], Buddy PRO [71], ELLI-Q [72], SOTA [73], and CANBOTU05 [74]);
- Large-sized robots are involved in healthcare environments to deliver drugs in various hospital wards (i.e., Relay [75], Tug T3 [76], Moxi [77], and CSJBOT [78]) or as a receptionist (i.e., CSJBOT robots [79,80,81] and ROBOVIE R3 [82]). This type of social robots also includes more realistic human-like devices involved in university research such as Sophia [83].

In this context, the social robot Sophia can be evaluated an illustrative case to clarify the adopted classification: the robot has been specially developed for university research, to experiment advanced artificial intelligence and even more human-like behaviors and appearance. Instead, its small sized version Little Sophia is involved in schools and educational environments with the aim of bringing together children and the social robot ecosystem. The ultimate purpose of Little Sophia is to firstly interface kids with coding and technology.

### 5.2. Appearance

#### 5.2.1. Description of the Uncanny Valley Phenomenon

Concerning the customization and the appearance of social robots, considerations about the Uncanny Valley phenomenon have been reported by Zia-ul-Haque et al. [84]. This research was based on the model proposed by Mori M. in 1970, who theorized that building robots too similar to humans evoke negative emotions and fear in people. Zia-ul-Haque et al. [84] describe an increase for the human positive response when the robots’ appearance is close to humans but the mechanical appearance of the systems is still recognized, and people are interested in having an interaction with them. The Uncanny Valley sets in when the mechanical aspect of the system is identified with difficulty, producing a feeling of unease and disturbance (lowest peak of the curve): robots as a result are unattractive and transmit mistrust, and people are reluctant to interact with them. The highest level of the curve has been reached when there is not theoretically a difference between the cyber entity and the human entity involved. Similar results have been collected to study the acceptance ratio depending on complexity of behavior. For instance, particular attention has been paid to implementing human skills for the gait of the robots. Amira et al. [85] proposed kinematic and dynamic models of a humanoid female prototype that is able to swing its arms in its gait. In this case, the solution proposed as an applicable model is able to ensure realistic and easy-to-control movements through separate consideration of lower and upper region of robot’s body. Moreover, the Uncanny Valley curve application field has been extended, and it can be adopted also to study more complex phenomena. For instance, Chung et al. [86], starting from the theory of Mori M., have investigated how robots’ level of anthropomorphism can affect privacy perception of users. Finally, the ultimate goal of research teams involved in social robots development is to realize devices capable of providing personalized assistance related to a very specific health context but characterized by a high degree of adaptability to different environments [87]. In this particular scenario, social robots have the ability to be perceived as effective social companions and engage in positive interactions with patients.

#### 5.2.2. Design Guidelines

In order to avoid the Uncanny phenomenon from an aesthetic point of view instead, a suggestion can be the adoption of 3D printing as a possible solution to design highly customizable social robots. An example of application of this approach is MaFaRo [88], a Many Faced Robot that can adopt different appearances according to the situation. It constitutes an optimal starting point to develop a low-cost head for social robots. Functionalities of conventional robotic head have been guaranteed, considering that the manufacturing process involves 3D printing with commercially available desktop printers. Another essential aspect of MaFaRo is its modularity, whereby each part may be plugged and played easily without using any screws. Finally, as mentioned above, designing robots’ appearance and behavior in order to avoid the Uncanny Valley effect may affect the emotions evoked in people by the systems. In this sense, technological improvements have been collected both from patent and bibliographic analysis. Stiehl et al. [40] have proposed the Huggable, a small bear robot involved in nursing homes and hospitals. An ad hoc designed sensitive skin has been implemented, in which tactile information from electric filed sensors and force sensors are coupled with signals from potentiometers and thermistors to obtain a specific somatic map. Hug-ability and affective touch have been demonstrated to be particularly relevant also in the patent field. Boyle et al. [89] have filed a patent that proposes the general structure for an huggable companion bot to assist individuals with mental illness. The system is composed of a first robust layer, capable of sustaining a composite clothing made of a memory foam layer and synthetic fur.

### 5.3. Degrees of Freedom 

Social robots are required to move nimbly in hospitals, households, homes, and other human environments. In order to be socially interconnected with people and to help them in daily life, they may have actuated joints (axes) to perform limbs’ motion, as well as motors that allow the eyelids to blink, gaze focusing on object and stakeholders, or other expressive motions. For social robots used in medical environments, two main categories have been theorized (Table 4):

- Highly actuated robots (HAR), that are cyber-physical systems in which several actuators and sensors (e.g., encoders) associated with the respective movable joints allow realistic movements. In this way, robots (humanoid or pet companion bots) are capable of better reproducing the motional behavior of the natural counterparts. For instance, NAO and PEPPER robots (Softbank Robotics) can be grouped in this section;
- Slightly actuated robots (SAR), that are cyber-physical systems in which some actuators, located on the robot in specific relevant points, allow carrying out significative and elementary movements, such as gaze-following or other human-like behaviors. For instance, JIBO [90] and VITA (InTouch Health) can be grouped in this section.

Slightly actuated robots are preferred embodiments for telepresence robots, nursing robots, and telemedicine robots; on the other hand, highly actuated robots are preferred embodiments for extremely realistic systems where the human-like aspect is essential. This last type of device may include commercial robotic platforms used for the research, such as Robovie-PC [91]. Every social robot must have actuators or servomotors in order to move and complete tasks. In their study [41], Bethel et al. used Dynamixel AX-12A servomotors on Therabot, an adaptive therapeutic support robot. The same devices were used by Salichs et al. [92] to develop the desktop robot Mini, which included them in the base, arms, and neck. As a result, it is possible to conclude that servomotors are interesting solutions for pet companion bots due to their low weight, high stall torque, and small size. This type of actuators is also used in some Robotis platforms and in general for four-leg and six-leg robots; they allow them to create motion even by servomotor chain (Daisy-Chain). They consist of a fully integrated DC motor with gearhead reduction and a controller with its own driver and network. These servomotors have been used in the Bioloid robot [93], a highly actuated robot (18 degrees of freedom), aimed at encouraging the elderly to accomplish 15 min of physical exercise. Other Dynamixel servomotor versions (MX-64AT and MX-28AT) have been implemented in the social robot “Arash” [32], whereas a geared brushed DC motor has been preferred for the mobile base. Regarding robots’ movements and navigation, telepresence and telemedicine robots are developed preferring wheels or omnidirectional bases, managed from motor control units. For instance, to minimize the magnitude of errors in tracking position and orientation of four-wheeled robots, Hasan et al. have proposed and evaluated a hybrid controller, combining the Backstepping-Type 2 fuzzy logic control and a social spider optimization [94]. It performs better than traditional controllers, thanks to the backstepping controller used to compute the torque on wheels, while the fuzzy logic control and the social spider optimization are used to compute gain parameters. Moreover, robots’ bases are typically surrounded by some bumpers, as in the Maggie robot [95] or Turtlebot 2 [48] for example, to prevent damaging collisions. Mobile bases may allow the connection to docking station that may be equipped with solar cells capable to provide electric energy to charge the entire system [96]. Moreover, specific motors may be implemented inside social robots to carry out specific tasks, such as motors for vibrational cues expected by the penguin companion bot developed by IKKIWORKS PTY LTD [97] or the therapeutic robot for elderly by UNIV HONG KONG POLYTECHNIC [98].

### 5.4. Sensors

Several different sensing devices are implemented in social robots in order to create an “analogic to digital bridge”, interfacing the cyber-physical systems to the environment and people. Sensors applied in this field can be considered as sensory organs and networks analogously to their human counterpart. In this way, robots not only resemble human entities and are able to relate with them, but with specific medical sensors robots can help the elderly, people with impairments, or children with cognitive disorders. In this section, the sensing devices have been grouped depending on their functionality: tactile sensors, audio sensors, video sensors, RFID devices, and sensors for medical purposes. All these macro-groups have been described and analyzed in the following subsections.

#### 5.4.1. Tactile Sensors

Tactile sensors constitute a composite and engineered skin that coats specific surfaces or parts of social robots. For instance, they are placed on the hands and arms of NAO and PEPPER robots allowing them to perceive the environment and touch objects and people. Networks composed of tactile sensors and pressure sensors are essential in order to obtain human-like behavior in robots, not only in humanoid systems during hugs for example, but also for pet companions such as PARO. In this case, several tactile sensors are located under a coating synthetic fur and give feedback to elderly people, when they touch or stroke it. The ultimate goal of providing this artificial sense is to realize a strong link between robots and humans. Commonly, this particular sensing device is implemented in social robots that adopt array structures of tactile sensors; in order to realize easily manufacturable and lower cost solutions, innovative flexible array-less tactile sensors have been developed [99]. Willemse and Van Erp described the importance of the touching phenomenon [100]: if the social touch has been carried out in a realistic precise way, the engagement between humans and machines can be improved as it happens in human–human interaction. In general, touches are complex actions that can reduce stress and anxiety and can enhance pro-social behavior. In order to assure a realistic touching phenomenon, Cabibihan et al. [101] have conducted some experiments on synthetic skin that may cover the forearm, palm, and fingers of a social robot arm. Thus, a power control scheme that can regulate the surface temperature of robots’ anthropometric regions such as human parts of the body (e.g., human arms and hands) has been proposed. Currently developed tactile sensors are constituted of thin polymer sheet with piezo-electric or piezo-resistive properties; moreover, they can be haptic devices with magnetic or optic properties, but in this case their implementation in social robotics is in the earliest stage. Mazzei et al. [102] have proposed an innovative technology based on stretchable silicon-made touch sensitive surface. This type of sensor records a pressure field which is elaborated by specific algorithms and exhibits a behavior similar to human mechanoreceptors.

#### 5.4.2. Audio Sensors

Audio sensors (microphones) are an essential part of social robots. Single-microphone systems have been gradually substituted by microphone array systems in which a plurality of sensors allow them to capture sound from the entire surrounding environment, in order to obtain a natural relationship with humans and eventually locate them only through their voice perception. Complex systems as Pepper [22,23,24,25,26] and NAO [16,17,18,19] present composite audio systems with four directional microphones; e.g., the tabletop robot KURI is equipped with a microphone array [103]. 

#### 5.4.3. Videos Sensors

A large number of different video sensors are implemented in social robots in order to record video parameters about the surrounding environment and humans. The main sensors used in social robotics are RGB cameras, thermal cameras, wide angle cameras, and three-dimensional cameras (stereo vision cameras and depth cameras). Different camera technologies allow for the obtaining of different results according to the image recognition algorithms. Depth cameras or kinetic sensors are constituted of infrared sensors coupled with a RGB camera where the data acquired by the second camera are represented in the three-dimensional space through proper mathematical models based on the emission and detection of an infrared ray. Examples of these technologies applied in social robotics are Microsoft Kinect [104,105] and Asus Xtion Pro Camera [106]. Stereovision cameras, based on the stereovision principle, require typically two or more sensors to obtain through the triangulation process three-dimensional information of a subject, as adopted, e.g., in Bumblebee2 FireWire camera [107]. The video sensors are not used just for facial and object recognition and mapping space; for instance in telepresence robotic systems, dedicated cameras may exist in order to provide images of humans during a remote conversation. Other types of devices, such as thermal cameras, have been adopted in human–robot interaction [108] or, specifically, to recognize the sign language, offering a new way to communicate with impaired people through a deep learning-based approach [109]. Commonly, the cameras are situated on the head or trunk of a humanoid robot or in specific and strategic points for non-humanoid systems. For instance, in the telepresence robot RP-VITA (In-touch Health), a camera is incorporated in the superior part of the larger display to record the patients and can be frontally rotated. The camera is mounted at the same level as an average height interlocutor in an upright position. Thus, in remote medical examinations, the face of involved people can be at the same height, generating an adequate interaction among them. Finally, QR code scanners may be useful for identifying patients and clinical history; an example of this solution is adopted in the Tico robot [110]. 

#### 5.4.4. Navigation Sensors

The navigation system of a social robot could be characterized by huge complexity, and it is realized with different sensors. These devices and the vision sensors, mounted in different positions, can work together, to assure collision avoidance, humans following a path, and autonomous navigation inside environments or aid in teleoperation during remote controlling of the robot. Many types of sensors may be adopted to allow robot navigation, such as ultrasonic sensors (SONAR), bumpers, LiDAR (Light Detection And Ranging) sensors, infrared sensors, IMUs (Inertial Measurement Units), and laser rangefinders. Inertial Measurement Units (IMUs) are used for the self-perception of a robot’s kinematics, e.g., in order to guarantee the equilibrium during their motion and working. IMUs are mainly composed of gyroscopes, magnetometers, and accelerometers [111]. Infrared sensors (IR) are used, for instance, in combination with sonar to improve robots’ capability to efficiently follow physicians in hospitals wards. In fact, ultrasonic sensors are adopted in some applications to map environments in a reliable and accurate way, while infrared sensors are used to define edges of an area. In other cases, IR sensors are mounted on a nurse robot and used in combination with a marking path line, on ward floor, to obtain precise following capability [112]. This last type of device may be affected by interference caused by sunlight; thus, sonar must be used to acquire precise information about the distance between robots and objects or humans, in outdoor and indoor environments. The constant innovation requested in this field has brought the application of new and more precise sensors such as LiDAR sensors. A Pepper robot application in assistive care scenario integrated with the AMIRO framework [26] utilizes LiDAR sensors to drive its obstacle avoidance module. The main purpose of the module is to detect obstacles, plan the path, and localize itself inside the environment. In order to perform this task, 360° RP1 LiDAR has been implemented with ROS, and it was connected to an acquisition board. Advanced solutions with LiDAR sensors allow an improvement in terms of embeddability and accuracy with the aid of SLAM algorithms integrated in a SLAM module, to provide a precise mapped environment for the robots. Most affordable solutions found in the patent literature [113,114] base avoidance obstacles on infrared sensors and sonars. In order to provide an optimal solution in terms of reliability and performances, Dongqing Du et al. have proposed a multi-sensor obstacle recognition system [115], adopting simultaneously sonar, IR, and LiDAR sensors. Table 5 reports the main feature of each sensor. A proper combination of these devices allows for the accurate mapping of wide-range environments, which reduces interference from lights and improves reliability, in accordance with desired applications.

#### 5.4.5. RFID Devices

Radio frequency identification devices (RFID) are sometimes implemented in social robots in the healthcare environment [116] and are based on the emission of radio waves at defined frequencies by emitters or solicited passive tags that have been read by scanners to extract information. The scanners are mounted on nursing robots and are able to read unique tags, related to the patient, in order to deliver specified drugs [117]. Moreover, radio frequency identification (RFID) can be used also in a localization system based on a Particle Filter (PF) and implemented in robots [118]. In this case, deployed tags, whose position is known, can provide 2D and 3D spatial and navigation information to robotic devices.

#### 5.4.6. Sensors for Medical Purposes

Several specialized sensors can be successfully implemented in assistive care environments and hospitals. The main application of these devices involves the use of nurse robots and telepresence robots that allow medical personnel to visit hard-to-reach people and in general monitor inpatient rooms and their bio-signals. According to the results of the current analysis, sensors for medical purposes may include blood pressure sensors [119,120], blood glucose sensors [121,122], blood oxygen saturation meters [119,123], temperature sensors [124,125], humidity sensors [125], and gas sensors [124]. Finally, it has to be reported that previous devices that normally constituted equipment for nurse robots, especially after the COVID-19 pandemic, can also be provided with disinfectant sprayers and UV-C light sterilizers [126].

### 5.5. Algorithms

#### 5.5.1. Data Elaboration

Several sensors extract and transform various analogic signals to digital parameters that are computed together to define the behavior of robots during human–robot interaction (HRI), allowing robots to fulfill tasks and to move around the environment. In this way, algorithms supported by the robots’ framework exploit the hardware calculation power to make robot autonomous during movements, in speaking and listening, as well as in people and object detection or mapping environments. In this sense, for instance, Ravindu et al. [107] have proposed an advanced mapping system able to identify objects and establish attributes to them, amalgamating spatial and objects maps to provide a better human–robot interaction. Matsuo et al. [127] have elaborated upon an innovative entropy method to avoid collisions for omnidirectional platforms. Their considerations are based on probability of collision; once the environment is mapped in blocks, each one has been related to a definite entropy, and higher values correspond to higher probability of collisions. Moreover, the movements of social robots, particularly inside crowded environments, are one of the biggest sources of concern. Thus, Correa et al. [128] developed a specific tracker, the Probability Hypothesis Density (PHD) filter, implemented in a system using a laser range sensor and capable of tracking people in a throng. Although algorithms are involved in every single feature of robots’ automaticity, localization algorithms are particularly under development, being the object of numerous studies. Systems based on Particle Filter (PF) localization filled the gap left by SLAM algorithm, solving positioning problem in an unknown environment. The PF framework can be also used in combination with Unscented Kalman Filter (UKF) to achieve self-localization, as demonstrated in trials executed using the NAO robot. In this context, Ullah et al. have proposed UKF and PF localization algorithms to enhance localization systems involved in robotic applications and wireless sensors networks [118]. 

#### 5.5.2. Artificial Intelligence

Algorithms and frameworks together constitute artificial intelligence, in which machine learning and deep learning are revealed to be promising techniques in the realization process of more realistic social partners for people, especially in the healthcare field. Concerning the machine learning relevance, some experiments have been conducted using the Support Vector Machine, Hidden Markov Model, and Artificial Neural Network, in order to provide human activity recognition [129]. Other machine learning techniques are implemented in facial expression recognition (FER) using artificial neural network architecture (e.g., with Autoencoders), transfer function, and a regression model to predict continuous emotions (e.g., with Support Vector Regressors) [130]. Social robots may have both the capability of recognizing emotions and expressing them. In this context, numerous scientific efforts have been made to develop robots capable of providing sympathy, an altruistic response to limit and reduce other’s pain and discomfort, rather than programming emphatic robots, which still remains the main limit in this field [131]. Furthermore, every emotional aspect of the robot or way of behavior in the interaction with people can be tailored to the user target, enhancing their mutual engagement. According to these considerations, multiple kinds of personalities have been developed in the analyzed social robots [132]. To investigate the connection between patent analysis and the scientific one, an interesting illustrative case is the patent [133] filed by Korea Ind Tech Inst. This patent is based on considerations made by C. Breazeal on his Kismet’s emotional space [35] and proposes an emotional robot, with a lip sync unit that is able to modulate mouth shape with the acoustic signals emitted. Concerning frameworks, they constitute the initial point to generate algorithms that can provide the robots with the ability to interact with humans. In particular, the CORTEX framework [134], Robocomp framework [135], UoA Robotic Software Framework [19], KnowRob [19], NAOqi [16,26], and AMIRO framework based on ROS (Robot Operating System) [26] have been highlighted as specific framework used in social robotics. The artificial intelligence inside a social robot is complex, and its structure is composed of many different types of algorithms, such as a speech recognition algorithm, autonomous navigation algorithm, gesture recognition algorithm, facial recognition algorithm, object detection algorithm, and emotion detection algorithm. Table 6 briefly collects the set of algorithms and AI services captured with the current analysis, depicting the main characteristics of each. On the results of the patents, analysis suggest that a lot of effort have been made to develop AIs capable of giving to social robots human behavior [63,68,136,137,138,139,140,141,142,143]. Thus, particular importance has been given to AI embedded in humanoid robots; these may be equipped with algorithms that allow realizing spatial-temporal and emotional reasoning. In this context, the main purpose of the patent filed by Singh et al. [136] is to build a cognitive structure able to show empathy, focus, and goal-reasoning skills. Empathy and the whole emotional sphere more in general have not to be intended just as output features provided by robots, but to generate bi-directional communication between social robots and humans, and several algorithms are employed in this field to interpret inputs from users. For instance, in chat robots, the adoption of a BERT pre-trained transformer model has been proposed for the emotions recognizing from texts [144]. Other types of algorithms, defined as text-to-speech algorithms, have been allowed to synthesize voices, specializing in transmitting robot-simulated emotions and instilling trust in users (i.e., patient, children, the elderly, and medical personnel). This is performed through different paralinguistic cues such as tones, accents, and vocal fillers. Finally, the complex system represented by HRI can benefit from the integration of all these types of algorithms, in a single more autonomous cyber entity, but it has to be mentioned that facial and movements cues are the most important in evoking users’ trust [145]. Thus, major efforts have to be made specifically for algorithms involved in these fields.

### 5.6. Connectivity and Hardware

**Figure 18:**
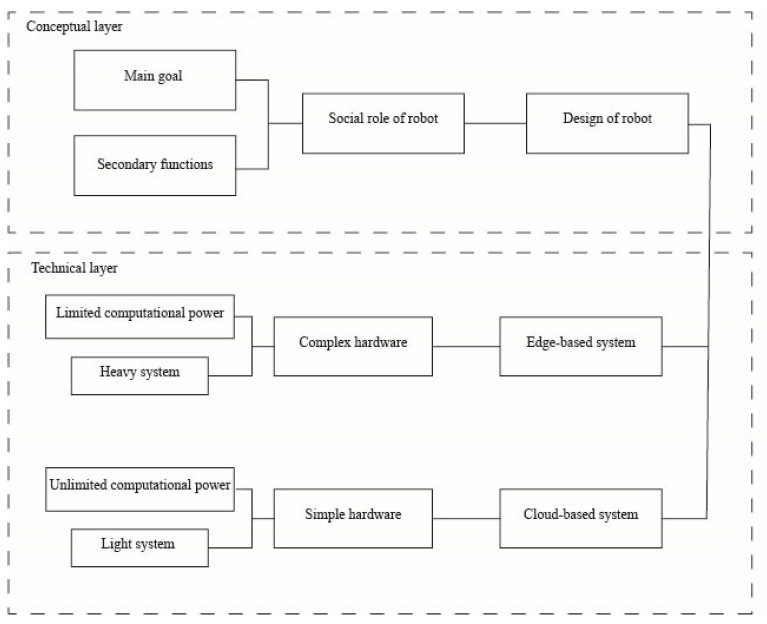
Factors that affect the final design of a social robot. Starting from the goals to be achieved, in the conceptual layer, a technical choice has to be made to locate the hardware for the computational power needed. Both these factors influence the final system.

The connectivity aspect is important in collaborative robotics when a large amount of data needs to be processed and it is not possible to keep the computational elaborator on board the machine for economic or technical reasons. In this case, various technologies and data transfer protocols are generally available over the air that enable the transfer of sufficient data for this type of operation. In order to carry out a virtual bridge between remote computers and robots in cloud-based systems, the most applied technologies are Wi-Fi (wireless connection), Bluetooth (wireless connection), ZigBee (wireless connection), and GPRS/3G/4G/5G (wireless connection). The first three types of connections are short range wireless technologies. While Wi-Fi and Bluetooth have become mass-adopted in human smart devices, ZigBee constitutes a less common mesh wireless protocol: it is standardized with IEEE 802.15.4-2003 and has found great use in IoT and advanced communications between smart devices (e.g., wearable devices [119]). The adoption of 5G transmission allows an increase of the amount of information that can be transferred wirelessly, reducing latency and incrementing the possibility of implementation of more intelligent systems [158]. Concerning edge-based systems, data collected and elaborated from the robots are exchanged with computers through Ethernet ports (wired connection) or USB ports (wired connection). Compared to a wireless connection, cabled connections have the advantage of transferring sensible data in a safe way, preventing possible leakage due to malicious attacks. Moreover, wires allow faster transfer speed, resulting in a better solution for a larger dataset or for fast applications. Starting from Intel Pentium-based computer mounted on the Security Warrior [147], the realism and the activities required to the social robots have become increasingly complex; then, the technological and scientific response to these needs has been more advanced and modular hardware. Complex architecture could require specific solutions; for instance, Meng et al. [159] present an example of an innovative edge-based service robot where a complex hardware system is composed of two main parts, and every algorithm is executed on specific modules according to the calculation power needed and the hardware architecture. The architecture is divided into two different systems: the lower microprocessor (STM32) has to be the core of the control system, meanwhile the upper microprocessor (Nvidia Jetson TX2 board) hosts the artificial intelligence based on deep learning technique. Another use case for the Nvidia Jetson TX2 is the social robot ARI [160], in which the GPU provides artificial intelligence to carry out self-learning and deep learning processes, and Intel i5 or i7 microprocessors are destined to computational operations. The literature presents some systems with high complexity, in which every part is specialized in a definite work, allowing for the building of smart robots capable of interacting with humans and the environment in natural and pro-social ways. Each hardware architecture is designed according to the goals that have to be achieved by the robots and the needed features related to the field of work. In general, it can be argued that parallel computing devices can be kept on board the machine for fast computation associated with social and security functions that require quick response, such as computer vision. All computational elements associated with mechanical functions must remain onboard the machine, whether they are strictly social, such as gestures, or useful for sociality, such as movement in an unstructured human social environment. Computational systems for more complex functions, such as verbal communication, must or may reside remotely for the time being, depending on the level of cognition to be incorporated into the robot (See in the Figure 18). 

### 5.7. Human–Robot Interaction

The social aspect of the robot is based on the depth and intricacy of its interactions with the human entity. Undoubtedly, engaging in close proximity and ensuring the safety of the human subject can promote a sense of positive social interaction. In this context, the role of actuators [161,162] and the implementation of secure compliant transmissions [163,164,165], as well as the implementation of control systems [166,167] and the utilization of intelligence to anticipate intentions by analyzing human movements [168,169], are of the utmost significance. Certainly the fundamental components of auditory and visual communication [170] along with the complex cognitive abilities required for comprehending language, visual stimuli, and audiovisual content serve as the foundation upon which the social robot can fulfill its intended purpose. In addition to possessing typical human capabilities, the robot also possesses the capacity to gather supplementary information through its on-board sensors, which are capable of perceiving audio and optical phenomena that surpass the typical human range. The Internet of Things (IoT) [171,172] enables the transmission of information from a sensorized environment to a robot. For instance, Zhao et al. [173] proposed a method, involving inertial measurement units (i.e., wearable sensors) positioned on patients body, to recognize a subject’s emotional state through convolutional neural network (CNN) inertial signal processing. Furthermore, wearable sensors facilitate the direct exchange of additional information between a human subject and the robot [174,175,176]. In the case of active devices [177,178], this exchange occurs in both directions. The availability of extensive information from curated databases or direct access to the Internet offers a valuable repository of knowledge that can be utilized for effective socialization [179]. Furthermore, the potential to utilize powerful parallel computing hardware platforms serves as a crucial factor in augmenting the analytical and generative capabilities of social robots [180]. Hence, it can be inferred that in the forthcoming era, the convergence of aforementioned technologies will culminate in the development of progressively complex and independent social robots. These robots are complex mechatronic systems [181] empowered by artificial intelligence [182] and possess the capacity to engage in meaningful communication with human counterparts, thereby imparting novel insights and perspectives that have the potential to enhance interpersonal connections. Consequently, this dynamic interaction will foster an engaging and continuously evolving relationship, owing to the remarkable capabilities inherent to the robot.

#### 5.7.1. Input Control Device

Social robots in healthcare and medical environments can be guided by expert operators through remote workstation and can be fully autonomous, such as the follow-robots that aid physicians and nurses in their daily work routine [183], or the control may be left to the patients themselves. Moreover, robots used in the neurorehabilitation field such as “Jessie”, do not require complex workstation to be controlled, since a simple application running on a tablet can manage them [103]. In addition, it has been demonstrated that social robots positively impact early-stage education; thus, many of them are involved in diagnostic trials and medical research programs involving children affected by neurological disease. In one case, the NAO robot (recognized as main teaching robot from subject matter experts) has been involved using the Wizard of Oz approach to investigate children with dysgraphia [21]. This type of control allows the physician or the educator to completely manage robot behavior. It is mandatory that device controls and degree of autonomy reflect caregiver, medical personnel, and patient needs. The way to realize the external controls can be very different according to the type of robot and to the type of interaction with humans. Inputs to the robot may be sent through a joystick: it can be virtual (on a screen) or physical or through a specific application with a GUI (graphic user interface). Different design solutions can be found in the literature to optimize the interaction; e.g., a patent filed by IROBOT CORPORATION and IN-TOUCH HEALTH describes some effective and simply interfaces [184,185]. Furthermore, headsets for brain–computer communication [16,22] and voice and gesture controls can offer innovative proposal to enrich the interaction. For instance, CHONGQING YOUBAN TECH CO LTD has filed a patent [123] in which an anthropomorphic system can recognize its specific partner through the voice and remain in an standby mode when other people try to interact with it. In other cases, social robots may be deployed in hospitals to assist the physician and not directly the patient. Biswas et al. have developed a touch-less nursing robot in which an integrated voice recognition module is connected to an Arduino MEGA [186]. The system can be controlled by a physicians’ voice to open his lockers or to be switched off. Moreover, RND GLOBAL CO LTD has filed a patent [122] for a wellness and human care robot able to provide several biological measurements (i.e., health state of urine test, blood glucose, blood pressure, body temperature, and body weight) and that can be controlled by elderly voices. Another field in which social robotics is especially useful and may be further implemented is childcare. In this context, parents or educators may be remotely involved in children’s activities thanks to the physical presence of a social robot that can be fully autonomous or in remote-control mode. In the first case, an HDM (head-mounted display) gives the parents the possibility of visualizing what the robot is looking at and what it is doing; in the second case, the robot can be guided, and an alter ego of the adult is provided by it [187]. Concerning the hospital environment, as proposed in the patent [188] filed by WEIN LEILA MAE, the medical robotic system has been programmed to operate in three modalities: the autonomous decision-making mode, providing output based on the robot’s embedded artificial intelligence; the doctor collaborative mode, in which artificial intelligence outputs are merged with medical staff experience; and the patient collaborative mode, in which the patient is interviewed by a robot interviewer to establish direct communication. Finally, the benefits provided by social robotics can be greatly enhanced by incorporating IoT units into systems, that can generate a dense network of information extracted from various sensors located on human users or in the surrounding environment. Different applicative examples of this technology can be found in the scientific and patent literature. For instance, Yoon Sang-seok et al. [137] proposed an IoT unit, embedded inside the robot, that collects significant parameters from a wearable device and a fine dust measuring sensor. Once information has been elaborated, an external air purifier is forced to work.

#### 5.7.2. Display and Touchscreen

Displays and touchscreens mounted on robots allow not only medical personnel but also children, the elderly, and impaired people to interact with the systems. Using a touchscreen display, it is possible to exchange information, preferences, and feedback with the cyber assistant. In this way, social robots become an essential partner during children’s education, assisting the elderly and impaired people that could present difficulties in establishing communication. For instance, Neef et al. [28] have proposed a novel health monitoring system, based on the Pepper robot, exploiting its frontal touchscreen, customized Android application, and sensing device. The user experience and the data acquisition system have been evaluated demonstrating that less previous expertise determines a higher level of robot acceptance by the users (e.g., elderly and children have a lower level of expectations). Thus, the importance of adequate people training in using health monitoring systems and other solutions have been cleared. Moreover, it has to be more deeply investigated whether a more transparent robot, in providing information on itself, can positively affect subsequent interaction with children. In this context, the training has been performed directly by social robots [189].

Analogously, displays play a fundamental role in telepresence robots, giving the possibility to show the faces of medical personnel during an examination or family members during a visit to a nursing home.

### 5.8. Impact of Ethics, Security, and Privacy on the Design of Social Robots

#### 5.8.1. Ethics in Social Robotics

In healthcare, social robots are artificial entities that can positively interact with the disabled, children, and the elderly, reducing the workload of nurses, physicians, and caregivers. Because of their artificial intelligence, they have a high level of interactivity. This feature allows them to be involved in multiple cases, and because many ethical issues are present, rules and design constraints will be considered. The main issue is the dehumanization of interactions in which vulnerable people are assisted by entities that are unable to experience emotions or understand the fragility of humans. In this sense, replacing human caregivers with care robots may be considered unethical. To combat this, developers have created algorithms that recognize emotions and facial expressions after they have been recorded as sensory data. This improves robot acceptance and usability, which is related to the systems’ natural cognitive ability. Similarly, in human–human interaction, artificial entities do not perceive people’s internal states but only their external representation. Furthermore, public trust influences the acceptance and subsequent deployment of social robots in assisting humans. This last factor improves if artificial systems increase the benefits provided to humans in terms of safety and well-being. The ethical design of social robots has become a prerequisite for their development. Some stakeholder research must be carried out at the very beginning of the design process. Thus, it may be discovered that human–robot interaction is preferred in some cases because robots have predictable behavior or because wider adoption of robots allows nurses and physicians to optimize their time in relevant activities for the benefit of the patient. The primary goal of creating a dependable and effective solution is to translate social norms, laws, and human behaviors into algorithms for intelligent artificial intelligence. Finally, to combat loneliness and dehumanization caused by robots, they can be used as companions to help people rather than as replacements for human caregivers [190]. 

#### 5.8.2. Privacy and Security

As the term “social” implies, social robots are used in public and private settings where they have close contact with people. Robots in healthcare may be used by the elderly, the disabled, children, or physicians. Various end users correspond to a variety of possible locations where robots may be involved. The need for data protection stems from concerns about protecting people’s privacy. In this regard, the law has promoted requirements and constraints for safely managing data recorded by social robots, such as video and clinical files, which frequently use wireless connectivity (e.g., cloud-based systems) to store information on servers. As a result, to realize systems that process personal data, “privacy by design” must be considered as a starting point in social robot development. In this context, blockchain technology has the potential to provide two critical benefits: privacy and security. Nonetheless, this is an innovative solution, and more research is needed to validate and implement it in real-world applications. In fact, blockchain technology is currently plagued by a trilemma of security, performance, and decentralization that cannot all be verified at the same time. The BlockRobot [191] proposed by Vasylkovskyi et al. exploits this technology to provide private data access in human–robot interaction, and Table 7 reports the main principles of this solution.

### 5.9. Guidelines for Social Robot Design in Healthcare Applications

Drawing guidelines for the design of social robots in medicine presents at least two problems: 1-one is related to the design of each social robot precisely because it is social, that is, intended to develop a relationship with the human; 2-a further one is determined by the medical-therapeutic context. In fact, designing social robots for medicine requires first and foremost the constant collaboration of the physician, who must indicate precisely the functions for which the machine is intended but must also take into account the type of human being with whom the robot must interact. The machine in this context must know how to interact with sick, weak, and fragile people, often infirm and sometimes disabled, discriminate in relation to whom the average standard of safety must be increased, and assure compliance with interactive rules not present in the non-medical intersubjective relationship. Therefore, the delineation of guidelines for social robots in the medical-therapeutic setting must give strong consideration to the two issues outlined above. Some more precise guidelines are given below in relation to methods and content that are appropriate to follow. In general, teaching a machine how to interact with a human means knowing how the human thinks, moves, and acts but also knowing the laws and ethical values that govern human social interaction. This has led and increasingly leads robotic engineering to work in interdisciplinary teams consisting of neuroscientists (psychologists, neurologists, sociologists, psychiatrists, etc.) and philosophers, ethicists, and jurists. However, things are more complex than what can be expected, because these teams not only face completely new scenarios but are tasked with teaching the machine a model of human beings that is not fixed and unchanging but gradually modified by the same interaction with the machine. The intersubjective relationship between humans and social robots has changed and is changing the human as much as the machine: while, on the one hand, the design of the social robot is modeled on the human, on the other hand, interaction with the social robot is changing the human, its sociality, its habits, its acting, and its very way of thinking [192]. In this way, the human that neuroscience and philosophy are called upon to describe today is a “mobile,” dynamic human, with a “fluid” physiognomy, and this dynamism and continuous transformation depend precisely on the proximity with the machine, which has changed rhythms and times, values, and habits of man. The man who relates to the machine today is no longer the same man of 50 years ago. The contribution of neuroscience and philosophy here is really decisive. No researcher or scholar today questions this, which therefore must be held firm: the design of social robots needs a multidisciplinary team [193] of experts who have in mind the processuality, the becoming of the human with whom the robot must interact. Robots and humans grow and develop together: knowing that there is this process and knowing it in its particularity is fundamental to effectively design a social robot. That is why, in these teams, philosophy plays a fundamental role: not only because, thanks to its holistic approach, it establishes the necessary interdisciplinary links, but especially because it is well aware of the dynamic and processual nature of the intersubjective relationship and also has the task of urging team members to keep it always in mind in the design. Let us now see how all this impacts when it comes to implementing a social robot in medicine. As anticipated, the figure of the physician is central and decisive: the team members must first listen to the robot’s purpose, applications, and functions. Here, it is precisely the purpose that determines the type of machine: if it is intended for heavy work (such as lifting patients for bed sores), then the robot will need size and mechanics of a certain type; if it is intended for pediatric settings, then physiognomy, expression, and tactility will be decisive: the robot will have to be smaller in size, having particularly developed facial expressions, to be capable of particular movements and sounds, presenting specifically designed tactility and sensoriality, to be equipped with profound biometric recognition capabilities, etc. The composition of the team is a particularly important part of the work, to which great attention should therefore be devoted. Having constituted the work team, an interactive working method must be chosen. One approach on which many authors agree is to place the user (human) at the center of the robot design activity, through a wide range of methodologies that are more or less assimilated or have some similarity to codesign [194], which consists of having users or user representatives actively participate in the definition of technical specifications in various ways, as we shall see later. From this point of view, the problem with the healthcare domain, as pointed out, for example, in [195], is that it is not so clear who the user is. In fact, the physician prescribes a drug or therapy to the patient, that is, uses a drug or therapy to treat the patient. Generally, the therapy or drug is delivered by a therapist or other health care personnel, who uses the doctor’s prescription to actually treat the patient. The patient must definitely enter into a relationship with the drug or therapy for the treatment to occur. The caregiver participates in the entire therapeutic process both emotionally, because he or she often has a personal relationship with the patient, and practically, by following the directions of the therapist and physician, even if he or she has no specific training in treating the disease. Again, the overall goal of treating patients is precisely why there are hospitals that use the entire therapeutic team for this very purpose. Identifying the user is not so easy, so it is more reasonably proposed to put the disease at the center of the therapeutic experience, in which all these individuals participate according to their specific roles. More generally, a usability-based design approach has recently been proposed for the healthcare environment [196], but this needs to be reworked to meet the social aspect of the robot we are focusing on. Usability and sociality must be held together precisely by placing disease at the center of the design. Disease is one of the attributes of the patient, as he or she remains a social human subject and should not be identified only with the disease. This attribute identifies a specific class of patients who are generally different from a standard healthy subject, even in the way they socialize, because of disorders that may involve the physical and especially the cognitive sphere. There is a pedagogy of relationship that the social robot must implement in order to contribute to the treatment of the disease because the patient is a weakened subject of the disease itself. This weakness is a reason for additional assurance toward the patient, which is, among other things, induced by the regulations in each state, particularly the fact that the robot may be perceived as a therapeutic tool, in which case it must comply with all regulations specific to these devices. Having defined the working method, which is always implemented iteratively through trial-and-error approaches converging on the optimal accommodation of the social robot in the context in which it is to operate, a major choice must be made. Because the patient is not standard and the diseases may be very different from each other, a decision must be made about whether to make a specific device for a disease or class of diseases or to make a multifunctional system. The approach changes radically: in the case of a multifunction device, the adaptation of the system is achieved a posteriori by analyzing the characteristics of the disease. In the case of the single-function system, on the other hand, the whole robot is designed to best fulfill a specific function. In both cases, it is necessary to identify all the subfunctions that the robot must perform by starting with a general definition and articulating it as specifically as possible. Functional analysis produces a set of qualitative attributes that the social robot must have that can be further identified quantitatively by technical specifications. Each attribute of the social robot can be associated with a module, i.e., an artifact capable of accomplishing the function, e.g., a module for verbal communication, another for vision, and yet another for nonverbal communication. To the extent possible, functional modularization is a simplifying factor in design in general and, especially in such a complex context as social robotics, can become a winning element in achieving a practical goal in a reasonable amount of time even by using modules previously made by the work team or other teams. In order to facilitate reusability and interoperability of the various modules, it is essential to interpose an abstraction layer (interface) around the module itself, whether it is computer-based, electrical/electronic, or mechanical in nature. Although it is theoretically possible to decompose a function into smaller and smaller parts, there is a stage below which pragmatically it is not possible to go because the various elements of the function must be highly correlated (integrated) and the communication time between the elements is excessively high compared to the time in which the function itself must be performed. Consider, for example, the realization of gesture movements in which various motors of a robotic arm and hand must move in a coordinated manner: one can articulate this function a lot, but the level of physical integration between the various functional elements must be high. When the response time of a certain function must be reduced, it is often important that the function be performed locally. If, on the other hand, the response time can be high, it may be decided to perform the function remotely, thus centralizing complex functions in a single piece of very powerful hardware, for reasons of economic efficiency but also for the objective practical limitations of footprint, weight, and energy delivery. This is the case for natural language analysis and the processing of appropriate responses also in natural language. We can thus distinguish on-board modules from in-cloud modules. A number of key characteristics present in most social robots are outward appearance, verbal communication, sensing, cognitive abilities (artificial intelligence), personality, and safety. The outward appearance must be evaluated very carefully, for example with regard to anthropomorphism, depending on the target subject the robot is to interact with and also considering cultural aspects, among which the choice of colors plays a relevant role, and white and light blue tend to be preferred. Facial expressions may also be a desired aspect, but at the expense of high complexity in motoring. Alongside nonverbal facial communication, gestures also play a reinforcing role and, for certain types of patients, such as the deaf, are strictly necessary. In general, verbal communication is the typical vehicle of human sociality, and because of the high complexity of this function, it is actually necessary to use an artificial intelligence system on remote hardware to process information, resulting in information security issues. The ability to process images and video in a real-world environment is another function that can be complex and require remote hardware, depending on how much information is to be extracted from the data. Other sensors can be used to capture health information from the patient and transfer it to a centralized database. The choice of a personality for the social robot is critical to achieving engagement and must clarify early on what the robot’s role is within the treatment team. The ability to move around in a partially unstructured hospital setting and in the presence of various individuals who are not necessarily professionals is an important lever for all of the robot’s social functions, but it represents an additional safety issue that must be addressed to avoid accidents. In general, the presence of sensor elements, moving mechanical parts, functions performed remotely, and the particular fragility of patients interacting with social robots force the designer to focus very carefully on cybersecurity aspects, more so than in cases of interactions with healthy subjects. In particular, issues concerning data privacy, authentication and access control, network security, firmware security, physical security, human factors, and regulatory compliance must be carefully analyzed. While all the aspects listed quite obviously lead toward the technical disciplines of cybersecurity, there is one that needs to be emphasized more than all the others: the human factor. In fact, because the social robot operates within human society, it is subject to social engineering and policy circumvention strategies that cannot be fully predicted, and, therefore, there can never be total security unless the risk factors are removed at the root and the social attribute is removed from the robot.

## 6. Conclusions

The current investigation was conducted by collecting data from three different sources (Scopus, Espacenet, and Google), with different levels of technological readiness (R&D, patents, and market). According to results, the introduction of artificial intelligence has undoubtedly accelerated the social robots’ evolution, which is also fueled by real-world issues such as the aging population and the COVID-19 pandemic. Indeed, robots have improved healthcare management in a variety of ways, from personal assistance to the elderly (e.g., nursing robots) to reducing anxiety and mental illness in people with disabilities (e.g., animal companion bots), as well as to assisting with children’s education.

## Figures and Tables

**Table 1:** TRL levels and related description.

TRL	Description
1	basic principles observed
2	technology concept formulated
3	experimental proof of concept
4	technology validated in lab
5	technology validated in relevant environment (industrially relevant environment in the case of key enabling technologies)
6	technology demonstrated in relevant environment (industrially relevant environment in the case of key enabling technologies)
7	system prototype demonstration in operational environment
8	system complete and qualified
9	actual system proven in operational environment (competitive manufacturing in the case of key enabling technologies or in space)

**Table 2:** List of all IPC groups present in the main dataset related to their frequency of occurrence. Concerning the first letter in the description of IPC number: index A identifies the “human necessities” field; index B identifies “performing operations” and “transporting” fields; index C identifies “textiles or flexible materials not otherwise provided for” field; index G identifies “instruments” field; index H identifies “electricity” field [1]. Subsequent indexes (numbers and letters) are a guide to more detailed information on patents and can be obtained from Espacenet website.

IPC Group	N. of Patents	IPC Group	N. of Patents	IPC Group	N. of Patents	IPC Group	N. of Patents
A61B5	181	G10L15	43	A61P25	21	G05B13	15
H04L29	162	B25J11	42	A63F13	21	G06N99	15
G06F3	137	G06T7	41	B25J19	21	G06T15	15
G06Q50	135	G05B19	37	G06F11	21	G10L25	15
G06K9	121	G06F15	36	C12M1	20	H04W8	15
G06F17	111	H04M1	36	C12N5	19	A61B90	14
G06Q30	101	H04W12	35	G01N1	19	A61P43	14
G06Q10	95	H04N7	34	H04B1	19	G01B11	14
G06F19	88	C12N15	33	H04N21	19	G09B5	14
G16H40	86	H04L9	33	H04W84	19	H04L5	14
G06F21	84	G06F1	31	A61K45	18	H04M3	14
G16H50	81	H04N5	31	B01L3	18	A61B6	13
G16H20	78	G06T19	30	C12N1	18	A63B71	13
G06F16	77	G01N35	29	G01S7	18	C07H21	13
G16H10	74	G06Q40	29	G06F40	18	G06F7	13
H04W4	73	G08B21	29	G06N7	18	G16H30	13
G06N20	68	G09B19	27	A61B8	17	A61B34	12
G01N33	58	A61K31	25	G06F13	17	A61H1	12
G06N5	58	B25J13	25	H04W88	17	A63B21	12
H04L12	58	G16H80	24	A61K9	16	G06F8	12
B25J9	56	G02B27	23	A61N1	16	G08B25	12
G06N3	54	G05D1	23	C07K14	16	G09G5	12
C12Q1	51	G06T11	22	G01N21	16	G16H70	12
G06F9	45	H04Q9	22	C07K16	15	H01L31	12
G06Q20	44	A61K39	21	G01S17	15	H04B17	12
						H04N19	12

**Table 3:** Robotic platforms relevant in social robotics for medical environment.

Type of Robot	Models
Humanoid robots	NAO [16,17,18,19,20,21]; PEPPER [22,23,24,25,26,27,28]; ROBOTIS OP3 [29]; INMOOV [30]; KIRO [31]; ARASH [32]; RAPIRO [33]; BRIAN 2.1 [34]; KISMET [35].
Pet companion -bots	NECORO [36,37]; PARO [9,38,39]; AIBO [10,36,39]; HUGGABLE [39,40]; THERABOT [41]; PLEO [39], SNUGGLEBOT [42], HAPTIC CREATURE [43,44], EDU’ [45], MAYA [46]
Telepresence robots	TURTLEBOT 2 [47,48,49]; CHICARO [50]; DALI [51]; INTOUCH VITA [52,53]; GIRAFFPLUS [54,55,56]; SIRA [57].

**Table 4:** Social robot classification. Highly actuated robots with DOF ≥14; Slightly actuated robots with DOF < 14.

Highly Actuated Robots (DOF)	Slightly Actuated Robots (DOF)
NAO (25)	RP-VITA (5)
PEPPER (20)	HUGGABLE (8)
INMOOV (47)	THERABOT (10)
ROBOTIS OP3 (20)	CHICARO (4)
KIRO (18)	EMIR (4)
ARASH (15)	RAPIRO (12)
KISMET (21—actuated face)	ARNA (7—robotic arm)
AIBO (22)	ROREAS (6)

**Table 5:** Comparison between LiDAR, infrared, and ultrasonic sensors.

Device	Advantages	Disadvantages
LiDAR	High detection accuracy	Long response time
		Expensive
Infrared sensor	Not expensive	Low accuracy
		Light provides interfaces
Ultrasonic sensor	High frequencies	Easily affected by noise
	Good directivity	Low accuracy
	Short wavelengths	Expensive
	Indicated for low-speed and short-distance measurements	

**Table 6:** Most frequently detected algorithms and AI service in social robotics, referring documents, and peculiar features.

Service	Peculiar Features
Amazon Polly Speech Synthesis [146]	Text to speech algorithm
Google Text-To-Speech [147]	Text to speech algorithm
Google Speech-To-Text [147]	Speech to text algorithm
PocketSphynx [19,148]	Speech recognition algorithm
IBM Tone Analyzer [147]	Emotions from text algorithm
YOLO V3 [25,26,134,149,150]	Object detection algorithm
Yolact ++ [151]	Object detection algorithm
SLAM [26,32,47,51,106,118,152,153,154]	Mapping and navigation algorithm
Baudi [146]	Face recognition algorithm
Procrob Functional [19]	Face recognition algorithm
Euclid [146]	Face recognition algorithm
Viola-Jones algorithm [155,156]	Face recognition algorithm
Google Dialogflow [19]	Speech and text machine learning service
IBM Watson Assistant [147,157]	Question answering computing system
Amazon Lex [19,146]	Deep learning service for natural speech recognition

**Table 7:** Main features of the BlockRobot.

Feature	Description
Correct data outline	Robotic events (i.e., videos or RFID tags read) are sent to BlockRobot. Human’s identities are pseudonymized and unrecognizable to robots.
Data persistence	Private data are hashed and stored on blockchain for verifiability. Private data can be stored in a ledger (off-chain device) external repository and encrypted. The solution proposed is a public key infrastructure. Robots’ signature needed to mark data recorded.
User interface and blockchain transaction	Intuitive “GUI” is provided. Possibility for the user involved to erase or access data. Digital signature by user is required. Blockchain’s block building is immutable and unchangeable. Data accountability is assured because on blockchain is present the history of all data.
Identity management by blockchain	Registration of users on the network through the BlockRobot API with subsequent smart contract authorization. Authentication of pre-registered users.
Core functionalities	Adding private data by hashing. RFID events and video recordings are both collected by the robot and processed by BlockRobot API. The private data are hashed to provide a new transaction to build the block for the chain. Private data are finally encrypted and stored off-chain. Accessing private data through smart contract that verifies the user identity, a new transaction is generated, and private data hashed before are accessible as the original one on user’s GUI. Deleting private data through smart contract transaction after verifying user’s identity.

## Data Availability

Data are available upon request to the authors.
